# Efficient training of mice on the 5-choice serial reaction time task in an automated rodent training system

**DOI:** 10.1038/s41598-020-79290-2

**Published:** 2020-12-21

**Authors:** Eszter Birtalan, Anita Bánhidi, Joshua I. Sanders, Diána Balázsfi, Balázs Hangya

**Affiliations:** 1 Lendület Laboratory of Systems Neuroscience, Institute of Experimental Medicine, Budapest, Hungary; 2 Sanworks LLC, Rochester, NY USA

**Keywords:** Neuroscience, Learning and memory, Motivation, Stress and resilience

## Abstract

Experiments aiming to understand sensory-motor systems, cognition and behavior necessitate training animals to perform complex tasks. Traditional training protocols require lab personnel to move the animals between home cages and training chambers, to start and end training sessions, and in some cases, to hand-control each training trial. Human labor not only limits the amount of training per day, but also introduces several sources of variability and may increase animal stress. Here we present an automated training system for the 5-choice serial reaction time task (5CSRTT), a classic rodent task often used to test sensory detection, sustained attention and impulsivity. We found that full automation without human intervention allowed rapid, cost-efficient training, and decreased stress as measured by corticosterone levels. Training breaks introduced only a transient drop in performance, and mice readily generalized across training systems when transferred from automated to manual protocols. We further validated our automated training system with wireless optogenetics and pharmacology experiments, expanding the breadth of experimental needs our system may fulfill. Our automated 5CSRTT system can serve as a prototype for fully automated behavioral training, with methods and principles transferrable to a range of rodent tasks.

## Introduction

In behavioral neuroscience, animal training requires a costly investment of work hours and resources. It is a non-trivial undertaking requiring human accuracy and persistence, constraining efforts to standardize and scale up behavioral experiments. There is an increasing need for high-throughput behavioral assays as systems neuroscience moves towards increasingly more complex behaviors, optogenetic manipulations and recording neural activity via electrophysiology or imaging in behaving animals^1^.

Systematic studies found that uncontrolled factors may have profound impact on the experimental results^2–4^. Moreover, potential subconscious biases of the experimenters may pose even larger problems than serendipitous differences. This is especially important in pharmacology and optogenetic experiments, where different handling of the treated and control groups, even in subtle ways, may lead to false results. Blinding the experimenter to the group identities averages such differences out as a consequence of the strong law of large numbers^5,6^; however, blinding is often not possible due to overt differences between experimental groups and convergence of the mean to the expected value may take prohibitively large samples^7^.

A few automated training systems have been developed for rodent behavioral tasks^8–15^, including the 5-choice serial reaction time task (5CSRTT)^16,17^, in order to standardize the training and reduce the effects of human factors and other random variables. While these systems provide means for large capacity automated training of rodents, most of them are customized to train a specific task variant, and/or contain expensive, proprietary components. For these reasons, automated behavioral training of the 5CSRTT task has not yet become widespread. Here we developed an affordable, open source, high-throughput automated training system for mice and demonstrate its use on an automated protocol of the widely used 5CSRTT assay^18–21^. We show that use of this Automated Training System (ATS) allows efficient training of mice while decreasing human labor expenditure due to the high number of trials performed daily. To improve upon existing systems described in the literature, we (1) provide an inexpensive, modular, open source training setup, (2) fully eliminate human interaction with the animals during training, (3) evaluate the effects of training breaks and transfer from automated to manual training setups, (4) demonstrate that automated training reduces stress compared to traditional training and (5) validate use of our training setup with wireless optogenetics and pharmacology experiments to increase the range of possible experiments the assay is immediately capable of.

## Results

### Stable performance despite decreased activity in the afternoon (middle of the light phase)

**Figure 1:**
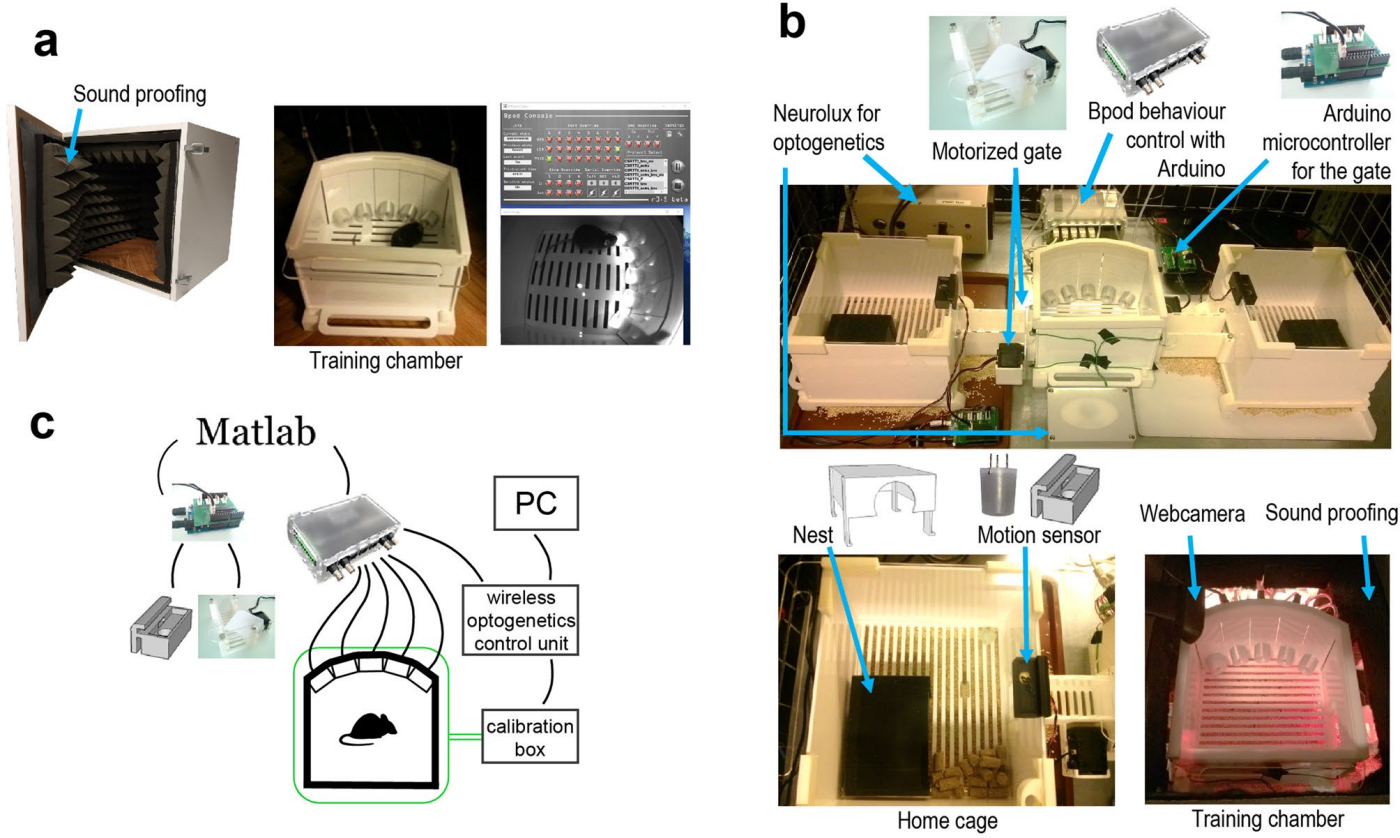
Behavioral setup. (**a**) Manual training setup. Left, the training chamber was placed in a sound attenuated wooden box (60 × 60 × 60 cm). Middle, the training chamber housed five behavior ports (Sanworks) each with an infrared photogate, a liquid reward tube and a visible (white) LED. Right, the behavior ports were controlled by the Bpod behavior control unit (Sanworks) during training (top), while the animal was monitored via a high definition camera (FlyCapture; bottom). (**b**) Automated training setup. The ATS (top) consisted of a training chamber (bottom right) identical to that of the manually trained animals except for the side openings, through which it was connected to home cages (bottom left) on both sides. The home cages were equipped with a 15 × 5 × 2 cm 3D-printed box filled with nesting material serving as nest for the animals, and a motion sensor (Panasonic) attached to the roof. The home cages were connected to the training chamber via tunnels blocked by Arduino-controlled motorized gates. The equipment for wireless optogenetics (Neurolux) and the Bpod behavior control unit were placed outside the ATS. (**c**) Schematic of the hardware-software connections of the ATS and wireless optogenetics apparatus. The Neurolux control unit and the water ports were controlled via direct connections to Bpod, whereas the motorized gate and motion sensors were connected to their corresponding Arduinos. The Bpod and Arduinos were connected to the computer via USB, and controlled by the same Matlab code ( available at https://github.com/sanworks/Pipeline_Gate and https://github.com/hangyabalazs/ATS).

**Figure 2:**
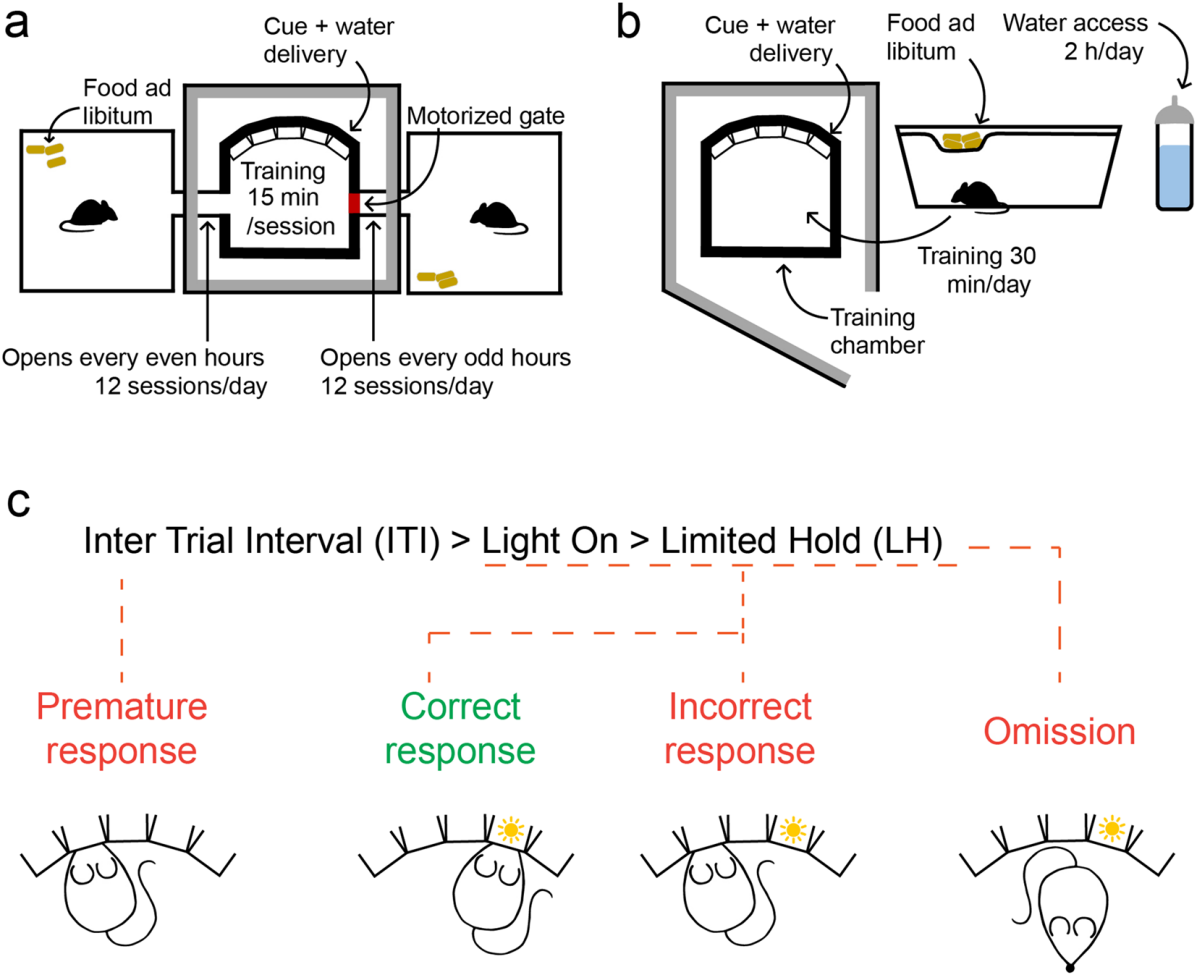
Training protocol (**a**) Schematics of ATS training. All animals had access to food ad libitum in their home cages, whereas they received water in the training chamber, accessible for 15 min in every two hours (free access to water at the beginning of each session and water rewards during training). (**b**) Schematics of manual training. Animals were kept in standard mouse cages with access to food ad libitum*.* Water was freely available for two hours/day. Mice were moved to the training chamber for 30-min training sessions daily, where they received additional water as reward, then moved back to their home cages. (**c**) Trial phases and possible outcomes of the 5-choice serial reaction time task.

We developed a fully automated, open source, modular training system, in which a training chamber was connected to two separate home cages, each housing a single mouse. Access to the training chamber was controlled by motorized gates, and mice were allowed to enter the training chamber based on a fixed, regular schedule of 15 min training every two hours (Figs. 1 and 2; Methods). The mice were kept on 12-h light/dark cycle, with light phase starting at 7 am.

**Figure 3:**
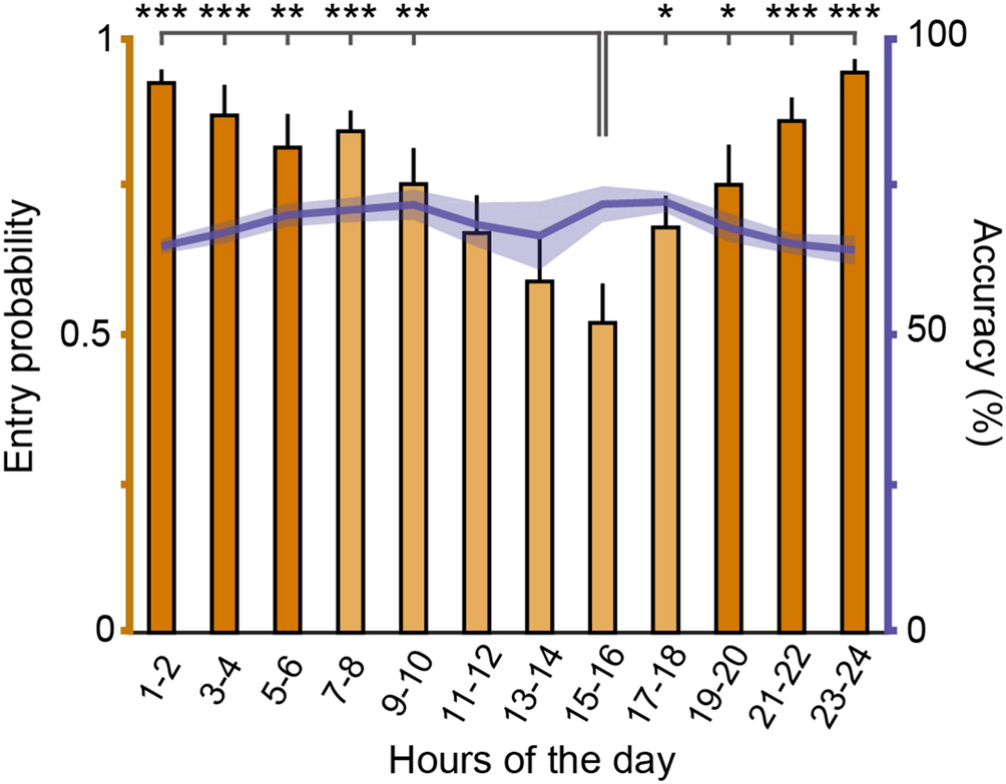
Mice were least active in the middle of the light phase but showed stable performance throughout the day. Activity (bar graphs, y axis on the left) was defined as the probability of mice engaging in a training session. Light phase (indicated by lighter colors) started at 7 am. Animals showed less activity in the afternoon (from 3 to 4 pm), but their accuracy (line plot, y axis on the right) was stable during the day. Bars and line plot show mean ± SEM. *, *p* < 0.05, ** *p* < 0.01, ***, *p* < 0.001, t-test; N = 16.

A group of 16 mice were trained on a 5CSRTT in the ATS (see Methods). Every two hours, the gate opened, giving mice the option to either enter the training chamber or skip a session. This allowed us to test whether mice show a natural preference for particular times of the day and whether accuracy in the 5CSRTT depended on what time the session was performed. We found that mice were least active between 3 and 4 pm, showing significantly lower probability of entering the training chamber (entry probability 3–4 pm, mean ± SEM, 0.52 ± 0.07; compared to 1–10 am and 5–12 pm, *p* and t-values for each time period between 1–10 am and 5–12 pm, respectively: *p* < 0.0001 , t = -6.39; *p* = 0.0001, t = -4.07; *p* = 0.0011, t = -4.03; *p* = 0.00039, t = -4.54; *p* = 0.0095, t = -2.97; *p* = 0.040, t = -2.25; *p* = 0.011, t = -2.89; *p* = 0.00039, t = -4.54; *p* < 0.0001, t = -6.03; Fig. 3) and omitting more trials when performing the task (mean ± SEM, 26.40 ± 3.92%, compared to 11 pm-6 am and 11 am-14 pm, *p* and t-values for each time period between 11 pm-6 am and 11 am-14 pm, respectively: *p* = 0.014, t = 2.81; *p* = 0.017, t = 2.71; *p* = 0.0042, t = 3.41; *p* = 0.034, t = 2.35; *p* = 0.0058, t = 3.25; *p* = 0.043, t = 2.23). Entry probability gradually declined from 9 am to 4 pm, then steeply increased to reach a maximum of 0.94 ± 0.02 (mean ± SEM) in the last hours of the day. While entry probability varied with circadian time, accuracy did not show significant fluctuations throughout the day (Fig. 3).

### More efficient training of mice in the ATS compared to traditional manual training

Mice were trained on the 5CSRTT, either manually or in the custom-developed ATS. In both systems, mice learned to respond to a brief presentation of a visual cue at one of five ports and reported cue detection by performing a nose poke in the illuminated port. Mice received water reward from the port for correct detections, followed by a variable inter-trial interval (see also Methods). During training, the light cue was shortened stepwise, creating stages of increasing difficulty, and mice had to reach fixed performance criteria to advance to the next training stage (Table 1)^20,22^.

**Table 1:** The 12 stages of difficulty of the 5CSRTT. The length of the visual cue (‘Light On’) and the additional response window (LH, limited hold) decreases through the stages. Criteria to advance to each stage are provided in the last row.

Stage	1	2	3	4	5	6	7	8	9	10	11	12
Light On (s)	30	20	10	5	2.5	1.25	1	0.9	0.8	0.7	0.6	0.5
LH (s)	30	20	10	5	5							
Criteria to reach the stage	-	≥ 30 correct answers	≥ 50 correct answers	≥ 50 correct answers > 80% accuracy	≥ 50 correct answers > 80% accuracy < 20% omissions							

We asked whether the training period required for mice to master the 5CSRTT could be shortened by training in the ATS, thus reducing experiment time, without increasing labor. This was addressed by comparing the behavior of mice (N = 16 in both groups) trained in the two different systems for an equal number of days. In contrast to the training schedule of the ATS described above, manual training was carried out in single daily sessions between 9 am and 12 pm and lasted approximately 30 min, to provide a comparison with common manual training protocols^20,22^. Mice were allowed to access water freely for an additional 2 h period per day (see also Methods, Figs. 1 and 2).

We also aimed to determine whether ATS-training is compatible with invasive experiments; therefore, we included a third group of mice (N = 7) that expressed a non-photoactive (‘control’) viral construct in basal forebrain cholinergic neurons and underwent stereotaxic implantation surgery of head-mounted LEDs for wireless optogenetic stimulation. These mice were photostimulated before the presentation of the light cue in 50% of the trials during ATS-training (see Methods).

**Figure 4:**
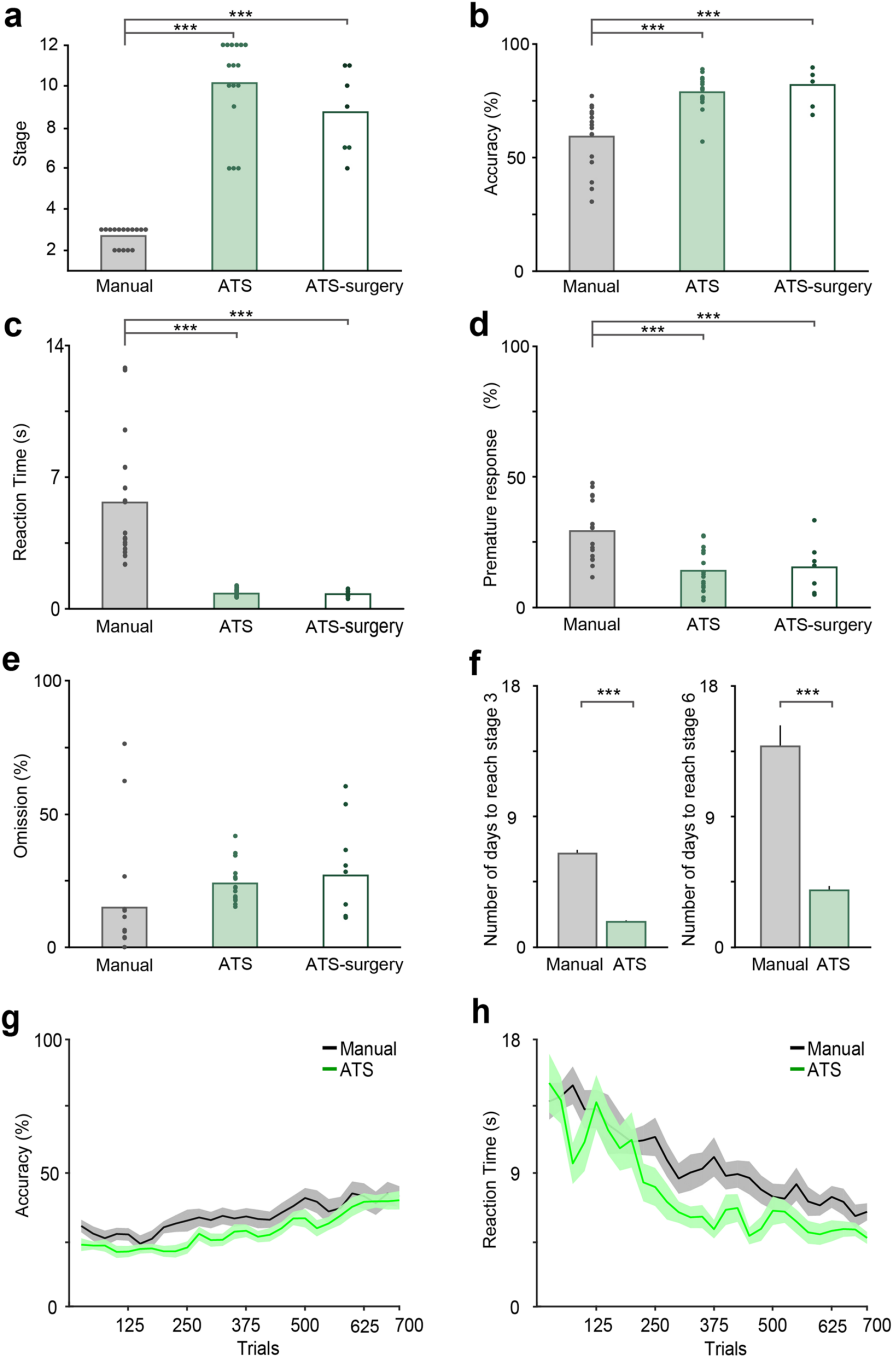
Mice reached better performance in the ATS in one week of training. (**a**–**e**) Behavior measures during the 7th day of training compared between groups of mice trained either manually, or in the ATS with (ATS-surgery), or without undergoing stereotaxic surgery. Bars, mean; dots, individual mice. Mice trained in the ATS reached higher stages (**a**), performed with higher accuracy (**b**), shorter reaction times (**c**), and performed fewer premature responses (**d**). There was no difference in omissions (**e**). Manual, N = 16; ATS, N = 16; ATS-surgery, N = 7. (**f**) Number of days needed to reach stage 3 and 6 in the manual setup and in the ATS. Stage 3, manual N = 13; ATS, N = 16; stage 6, manual N = 8; ATS, N = 16; two manually trained mice failed to reach stage 6 in 21 days of training (not included). (**g**–**h**) Accuracy (**g**) and reaction time (**h**) calculated for the first 700 trials of training (in 50 trial-windows with 50% overlap) for manually (grey) and ATS-trained (green) mice; lines and error shades represent mean ± SEM. Manual, N = 16; ATS, N = 16; ATS-surgery, N = 7. ***, *p* < 0.001; one-way ANOVA and repeated-measures ANOVA (**g**–**h**).

Learning performance was compared after one week of training (Fig. 4). Specifically, the average of a theoretical maximum of 12 sessions in the ATS on day 7 was compared to the single manual training session on the corresponding day in the traditional setup. Mice advanced through the twelve classical training stages of 5CSRTT (Table 1)^20^ automatically based on their performance; therefore, it was possible to compare the training stages they reached by the end of one week. Six of the ATS-trained animals reached the highest, twelfth stage, and all of them advanced beyond stage 5. In contrast, manually trained animals did not pass the third stage by the end of the week, achieved by 69% of the animals. Thus, we found that mice reached higher stages in the ATS when trained for one week (stage, F_2,36_ = 81.40, *p* < 0.0001; one-way ANOVA; Fig. 4a). Of note, this was also true for the ATS-trained group that had undergone implantation surgery (*p* = 0.00012, Newman–Keuls post hoc test).

Beyond reaching higher stages in the ATS, we found significant main effects between the three groups in their accuracy (correct trials divided by the sum of correct and incorrect responses, F_2,36_ = 16.95, *p* < 0.0001), reaction times (time between cue onset and correct response, F_2,36_ = 23.31, *p* < 0.0001), percentage of premature responses (ratio of prematurely terminated to all trials, F_2,36_ = 7.76, *p* = 0.00032), but not in percentage of omissions (ratio of omitted to all initiated trials – this excludes prematurely ended trials^23^, F_2,36_ = 2.47, *p* = 0.1; one-way ANOVA, Fig. 4b–e). Post-hoc tests revealed that ATS-trained mice were significantly more accurate than manually trained animals, regardless whether implantation surgery was performed before the ATS training (ATS, *p* = 0.00026; ATS-surgery, *p* = 0.00017, Newman–Keuls post hoc test; Fig. 4b). No significant difference in accuracy between the implanted and intact mice trained in the ATS was found (*p* = 0.5, Newman–Keuls post hoc test).

While the time windows in which mouse responses to cue stimuli were accepted varied across training stages, all mice had at least 5 s to perform a correct response. Mice trained in the ATS typically performed fast responses (mean ± SEM, 0.81 ± 0.04 s) with significantly shorter reaction time than manually trained animals (mean ± SEM, 5.65 ± 0.84 s; ATS, *p* = 0.00013; ATS-surgery, *p* = 0.00014; implantation surgery did not lead to a difference in reaction times, *p* = 0.98; Newman–Keuls post hoc test, Fig. 4c). We also found that ATS-trained mice performed fewer premature responses (ATS, *p* = 0.0031; ATS-surgery, *p* = 0.0026; no difference between the ATS groups was found, *p* = 0.76; Newman–Keuls post hoc test; Fig. 4d).

We next compared the time it took for mice to reach stage 3 (the highest stage reached in one week during manual training) and found that mice trained in the ATS reached this stage significantly earlier that manually trained mice (manual, 6.46 days; ATS, 1.75 days; *p* < 0.0001, one-way ANOVA). We found similar results when we compared the time it took for mice to reach stage 6, enabled by experiments in which mice were trained for longer periods of time (manual, 13.88 days; ATS, 3.94 days; *p* < 0.0001, one-way ANOVA; Fig. 4f).

In summary, after one week of training, ATS-trained mice reached higher training stages, performed with higher accuracy and shorter reaction times, and had fewer premature responses, indicating that ATS-training can decrease the time investment needed to achieve high performance. Although more intensive manual training may also decrease the length of the training period, this would be achieved at the cost of increased human resources. In contrast, ATS training reduces the number of days to training criterion, while also substantially decreasing total working time.

### Analyzing the impacts of training intensity in the ATS

To better understand the source of the above training benefits of the ATS, we performed two additional analyses of behavioral performance. First, to dissociate whether better performance of ATS-trained animals was due to a steeper learning curve, the higher number of trials performed (ATS, mean ± SEM, 753 ± 18 trials/day; manual, mean ± SEM, 155 ± 6 trials/day) or a combination of both, we compared performance improvement and reaction time in two training groups (manual, ATS) for the first 700 trials completed, calculated in 50-trial sliding windows (50% overlap; Fig. 4g–h). We found similar learning curves (group, F_1,30_ = 4.02, *p* = 0.054; time, F_27,810_ = 11.51, *p* < 0.0001; time x group, F_27,810_ = 0.66, *p* = 0.91; repeated-measures ANOVA, Fig. 4g) and similar reaction time curves (group, F_1,30_ = 8.54, *p* = 0.0065; time, F_27,810_ = 14.31, *p* < 0.0001; time x group, F_27,810_ = 1.04, *p* = 0.40; repeated-measures ANOVA, Fig. 4h) in the two groups when plotted as a function of completed trials, suggesting that the ATS-trained animals showed increased performance compared to traditional manual training due to the large number of trials mice completed during the 12 possible daily sessions. When we compared the number of trials required to reach stage 3 or 6, there was no major difference between training protocols, although mice trained in the ATS reached stage 3 in significantly less trials (F_1,27_ = 19.19, *p* = 0.00016; one-way ANOVA; Supplementary Fig. S1a–b).

Second, to learn whether the training parameters showed any differences within the same training stage, we compared the performance of manually (N = 13) and ATS-trained (N = 16) groups at stage 3 (the highest stage manually trained mice reached in 7 days; Supplementary Fig. S1c–f). We found that at stage 3, animals in the manual setup performed with higher accuracy (F_1,27_ = 20, *p* = 0.00012 ; one-way ANOVA), but with similar reaction times (F_1,27_ = 0.36, *p* = 0.55; one-way ANOVA), while performing fewer premature responses (F_1,27_ = 17.53, *p* = 0.00027; one-way ANOVA). There was no difference in the proportion of omissions between the groups (F_1,27_ = 1.47, *p* = 0.24; one-way ANOVA). Since ATS-trained mice reached stage 3 by day 2, compared to day 7 in the manual setup, these stage 3 results are consistent with the expected performance benefits of several additional days of memory consolidation during manual training^24^.

Thus, we propose that ATS-trained mice benefited from more trials performed, while manually trained mice may have benefitted from more time available for offline memory consolidation between sessions and advancement of stages. However, despite the different progression through training stages, the ATS pipeline enabled significant time savings and thus more efficient experimentation.

### Training breaks introduce a transient drop in performance, but ATS-trained mice maintain benefits over manual training

Optimal design of electrophysiology or optogenetic experiments often requires a training period, followed by surgery and recovery, after which training is resumed, combined with recording or manipulating a selected set of neurons. Typically, this leads to a transient drop in performance. Therefore, we sought to determine whether such a protocol would cancel some of the benefits of the ATS. Our main goal was to evaluate how ATS-training compares with classical manual training protocols within comparable training duration.

**Figure 5:**
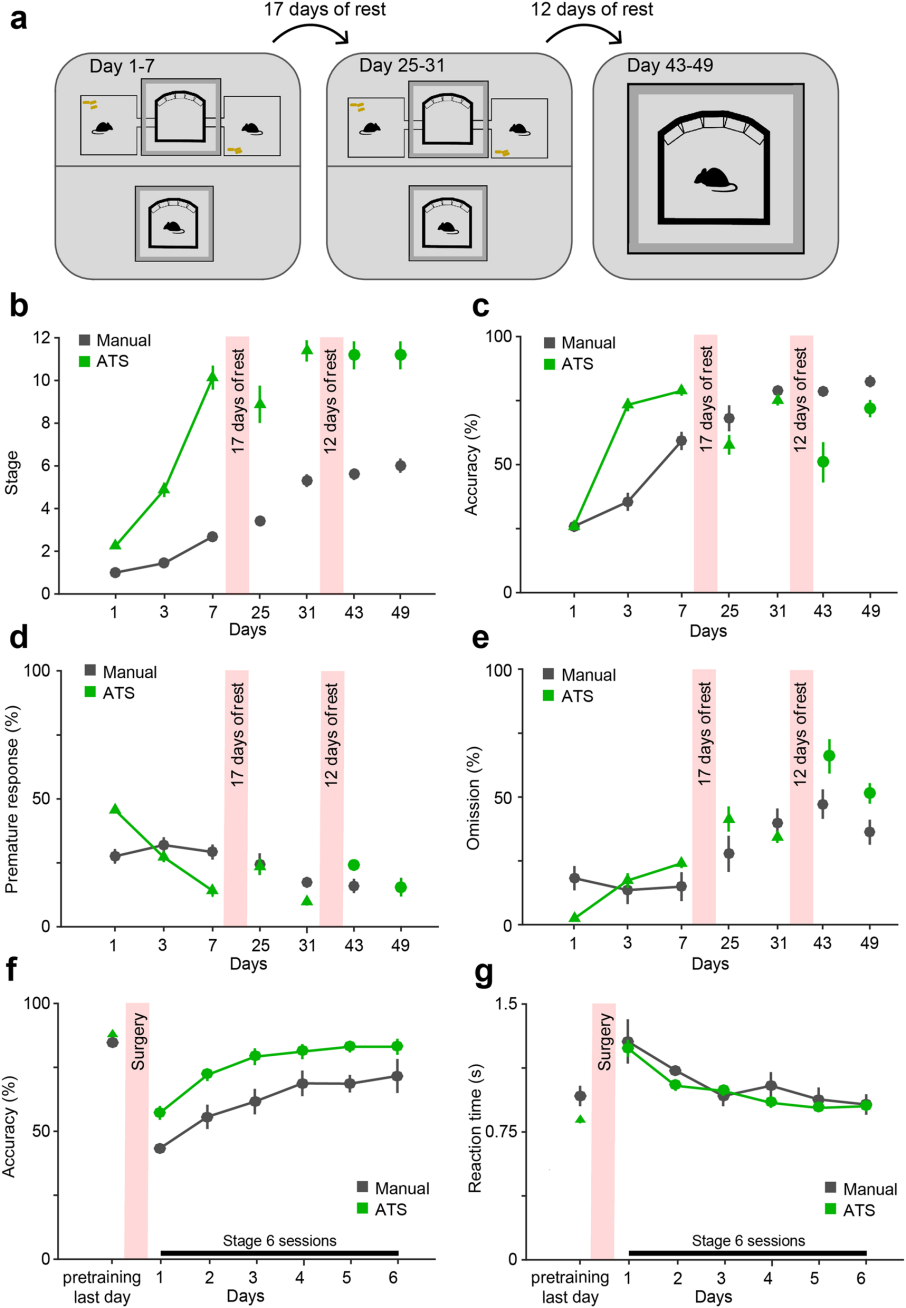
Effect of training breaks on performance. (**a**) Schematics of the experiment. One week of training was followed by a 17-days-long training break, after which mice resumed training from their previous stages in the same setup for another week. Following a second break of 12 days, all mice were transferred to the manual training setup. (**b**–**e**) Comparison of stage (**b**), accuracy (**c**), premature responses (**d**) and omissions (**e**) between the manual and ATS groups. After both breaks, ATS-trained animals showed a transient lapse in performance compared with manually trained mice, which difference disappeared during one week of training. Manual, N = 16, 10, 10, ATS, N = 16, 8, 6 at training week 1,2 and 3, respectively. (**f**–**g**) Accuracy (**f**) and reaction time (**g**) after surgical implantation of optic fibers. Mice were pretrained to stage 6 either manually (black) or in the ATS (green) and trained manually after the surgery. The two groups did not differ in their post-surgery recovery of these behavioral measures (no significant time × group interaction, *p* > 0.05, repeated-measures ANOVA). Manual, N = 5; ATS, N = 14. All values represent mean ± SEM.

Therefore, we measured the efficiency of both manual and ATS training interrupted by pauses (Fig. 5a–e). First, a 1-week training period was performed as shown previously (Fig. 4), then a 17-days pause was introduced to model training breaks imposed by surgery and recovery (manual, N = 10; ATS, N = 8 mice). After the pause, training was resumed at the stage mice had reached by the end of the first week of training. Compared to day 7, ATS-trained mice showed a transient decrease in accuracy and increase in prematurely terminated trials after the pause (accuracy, *p* = 0.012, premature responses, *p* = 0.025, omissions, *p* = 0.025, Wilcoxon signed rank test between day 7 and 25 in the ATS; comparing difference of post-pause and pre-pause values for ATS vs. manual training, accuracy, *p* = 0.045, premature responses, *p* = 0.015, omissions, *p* = 1.00, Mann–Whitney U-test; Fig. 5c–e). However, these changes vanished, and behavioral measures reached pre-pause levels within an additional week of training (by day 31). This transient performance drop may be explained by the fact that manually trained animals only reached stage 3 by day 7, thus resumed training at an earlier training stage compared to ATS-trained mice, which had been trained to stage 8 on average before the pause (Fig. 5b; see also below).

In practice, electrophysiology experiments may require large implants, head stages and tethering of mice to data acquisition equipment during behavioral training, precluding the use of ATS. Nevertheless, ATS may still speed up such experiments by allowing rapid pretraining of mice before implantation. In this case, mice are switched from ATS to manual training. We wondered whether such a change of environment could lead to a drop in performance, if mice failed to generalize over the training systems. To address this, we introduced a 12-days-long (from day 31 to day 43) second break of training with the same mice (manual, N = 10; ATS, N = 6 mice), after which ATS-trained mice were transferred to the manual training setup (Fig. 5a–e). Accuracy, omissions and premature responses followed the same general pattern as for the first break: ATS-trained mice showed a transient performance drop (accuracy, *p* = 0.046, premature responses, *p* = 0.028, omissions, *p* = 0.028, Wilcoxon signed rank test between day 31 and 43 in the ATS; comparing difference of post-pause and pre-pause values for ATS vs. manual training, accuracy, *p* = 0.026, premature responses, *p* = 0.011, omissions, *p* = 0.0079, Mann–Whitney U-test; Fig. 5c–e), but recovered after one week of training, reaching performance levels comparable to that before the pause. In sum, ATS-trained mice maintained their superior behavioral performance in terms of training stages reached, through the entire period of training, including post-break periods (Fig. 5a). The introduced training breaks led to a transient performance drop in ATS-trained animals, which was fully abolished by one week of re-training, showing that the ATS-trained mice maintain training benefits over their manual counterparts when training pauses have to be introduced, as in experiments using invasive techniques that require surgery.

### Recovery of behavioral performance after surgery

In the previous experiment, we compared ATS and manual training in an equal number of training days. This led to higher training stages of the ATS-trained mice at the times when the breaks were introduced (first pause: manual, stage 3; ATS, stage 8; second pause: manual, stage 5; ATS, stage 11). We wondered whether the more pronounced performance change of ATS-trained mice caused by these pauses were due to this stage difference. Therefore, we trained mice to the same stage in the two systems in a separate experiment and tested how mice recovered training performance after fiber implantation surgery.

First, the animals were pre-trained until they reached stage 6 (see Methods) with either the manual (N = 5) or the ATS (N = 14) protocol. No major differences in accuracy or reaction time were observed between the groups after pretraining (see ‘pretraining, last day’ in Fig. 5f–g). Then, after viral injection and surgical implantation of bilateral optic fibers and a subsequent recovery period of 3 weeks, all animals resumed training in the manual setup at stage 6, while two optic cables were connected to their implants at the start of each session to mimic tethered optogenetic experiments. The surgery and the pause in training caused a similar drop in both groups’ performance, but after a few days, their accuracy and reaction times reached the same level as on the last day of pretraining (Fig. 5f–g). Accuracy was higher in the ATS group after the pause, whereas there was no substantial group difference in reaction time, proportion of omissions and premature responses (accuracy: group, F_1,17_ = 16.26, *p* = 0.00086; time, F_6.102_ = 27.96, *p* < 0.0001; time x group, F_6,102_ = 1.19, *p* = 0.32; reaction time: group, F_1,17_ = 5.08, *p* = 0.038; time, F_6.102_ = 14.85, *p* < 0.0001; time x group, F_6,102_ = 0.72, *p* = 0.64; omissions: group, F _1,17_ = 1.2, *p* = 0.29; time, F_6,102_ = 14.32, *p* < 0.0001; time x group, F_6,102_ = 3.34, *p* = 0.0046; premature responses: group, F _1,17_ = 0.67, *p* = 0.42; time, F_6,102_ = 27.95, *p* < 0.0001; time x group, F_6,102_ = 3.33, *p* = 0.0049; repeated-measures ANOVA, Supplementary Fig. S2).

Thus, we found no substantial differences between training groups when surgery was performed on mice trained to the same stage. This experiment also demonstrated that animals pre-trained in the ATS were able to acquire the task quickly and efficiently, and seamlessly transfer to manual training even after the negative effects of surgery and training pauses, performing similarly to mice pretrained in the manual protocol.

### Training in the ATS causes less stress for the animals

**Figure 6:**
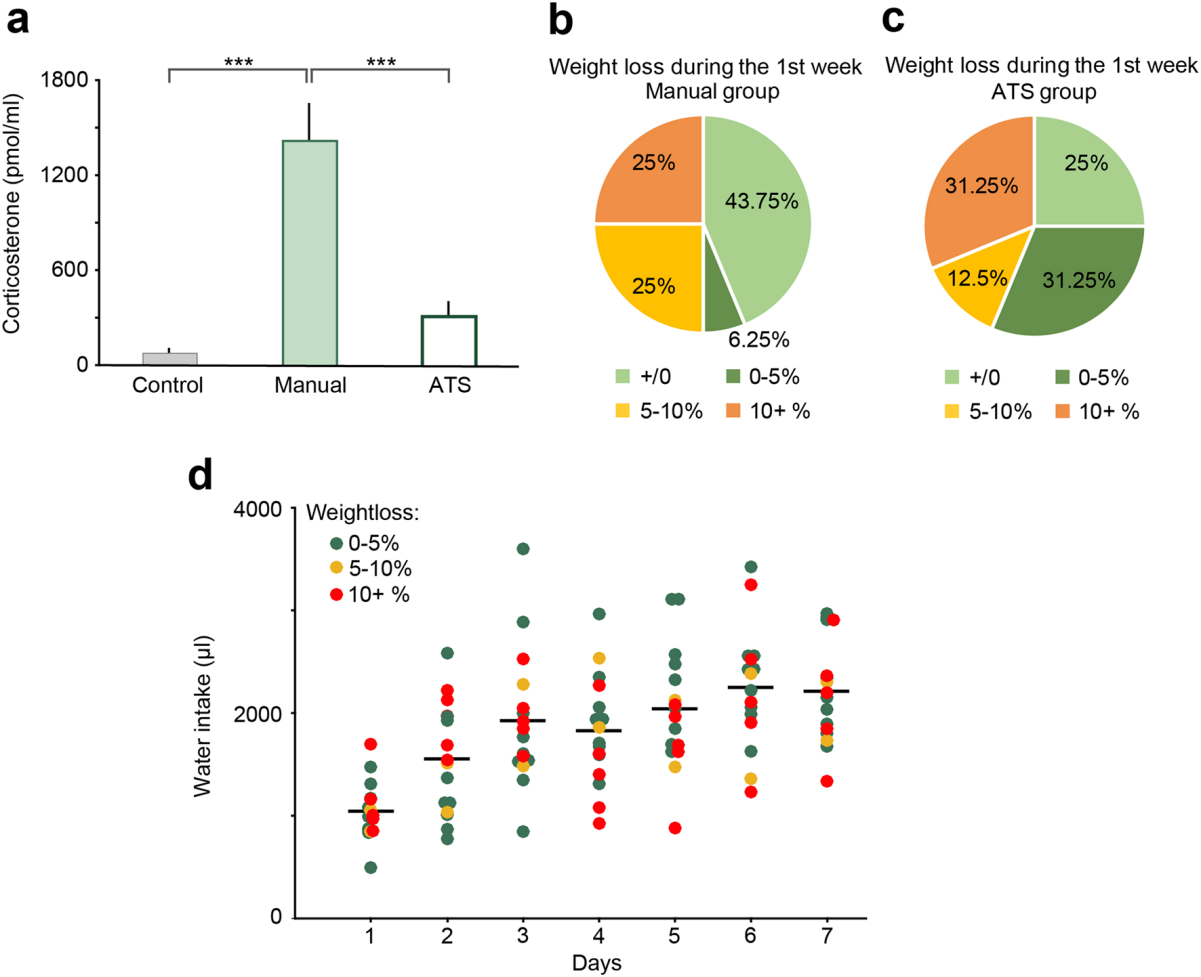
The effects of manual and ATS training protocols on stress hormone levels, bodyweight and water intake. (**a**) Blood corticosterone levels were higher in the manually trained mice when compared to the control or the ATS trained mice. There was no difference in blood corticosterone levels when comparing the control and the ATS trained mice. *** *p* < 0.001, one-way ANOVA; control, N = 6; manual, N = 6; ATS, N = 6. (**b**–**c**) Changes in body weight between training day 1 and 7 in the manually and ATS-trained mice. In the ATS group, more animals lost less than 5% of their bodyweight (including animals that gained weight) than in the manual group. (**d**) There was no correlation between bodyweight change and water intake in the ATS-trained mice. Dots represent the water intake of individual mice color-coded according to their bodyweight-change after one week of training. Lines represent average water intake. Manual, N = 16; ATS, N = 16.

We hypothesized that ATS may cause less stress for mice, since they are not handled or in any other way disturbed by lab personnel, and are free to decide whether to engage in the training at each scheduled opportunity^25–27^. To test this, we collected blood samples and measured changes in the concentration of corticosterone, the main glucocorticoid hormone regulator of stress responses in rodents^28–31^. After the last behavioral session on the 7^th^ day of training between 9 am and 12 pm, mice were allowed (ATS-trained) or transferred (manually trained) to their home cages for 10 min, after which mice were transferred to a separate room for blood sample collection (see Methods). Mice consumed comparable amounts of water in the ATS and manual setups before hormone testing. We found a significant main effect of corticosterone levels between groups (F_2,15_ = 22.81, *p* < 0.0001, one-way ANOVA; Fig. 6a). Post hoc tests revealed that corticosterone concentration of the manually trained mice (N = 6) was significantly higher than that of the control (N = 6, *p* = 0.0002) and the ATS-trained groups (N = 6, *p* = 0.00026), while the ATS-trained group did not show a significant difference versus the control group (*p* = 0.27, Newman-Keuls post hoc test; Fig. 6a). These results demonstrate that automated training causes less stress to mice compared to manual training and handling, despite the larger number of sessions, more completed trials and longer cumulative training time in the ATS.

Finally, we monitored the weight of mice during training. Water restricted mice typically show a mild weight loss after the first week of training. We did not find a significant difference between weight changes in the ATS compared with manual training (F_1,30_ = 0.74, *p* = 0.40, one-way ANOVA), although more animals tended to show < 5% weight loss in the ATS (Fig. 6b–c). Surprisingly, weight changes did not show an obvious correlation with the cumulative water intake of the animals (*p* = 0.2, R = -0.34, Fig. 6d).

### The effects of different water restriction schedules on manual training

Finding that weight loss was < 5% in many animals raised the question whether a stricter water restriction schedule would increase the efficiency of manual training^32^. To test this, we trained a group of animals with a modified manual protocol, allowing them to consume 1.8 ml water per day, which matched the average daily water consumption of mice in the ATS (controlled water consumption group, manual CWC, N = 6).

After one week of training, this manual CWC group reached an intermediate training stage (stage 6 on average) compared to our previous manual protocol and the ATS training, with accuracy and reaction times similar to the ATS group, but a higher number of premature responses (stage, F_2,35_ = 86.08, *p* < 0.0001; manual, *p* = 0.00014, ATS, *p* = 0.00012; accuracy: F_2,35_ = 17.81, *p* < 0.0001; manual, *p* = 0.00016; ATS, *p* = 0.78; reaction time: F_2,35_ = 21.53, *p* < 0.0001; manual, *p* < 0.001; ATS, *p* = 0.78; premature response, F_2,35_ = 10.25, *p* = 0.00031; manual, *p* = 0.51; ATS, *p* = 0.0073; one-way ANOVA, Newman-Keuls post hoc test; no difference in omissions, F_2,35_ = 1.53, *p* = 0.23; one-way ANOVA; Supplementary Fig. S3–4).

However, when we compared the weight changes of the three groups, although we did not find a significant main effect of the training group (F_2,35_ = 1.78, *p* = 0.18; one-way ANOVA Supplementary Fig. S3f.), two thirds of the animals in the manual CWC group lost more than 10% of their body weight (Supplementary Fig. S3g).

In conclusion, controlled water consumption improved the performance of manually trained animals compared to the original manual group that had 2 h of free access to water per day; however, these mice did not reach the same training stages as the ATS-trained animals. At the same time, they tended to lose more weight, raising caveats about attempts to improve learning speed by stricter water consumption schedules.

### Optogenetic and pharmacological manipulations of the cholinergic system impaired performance

**Figure 7:**
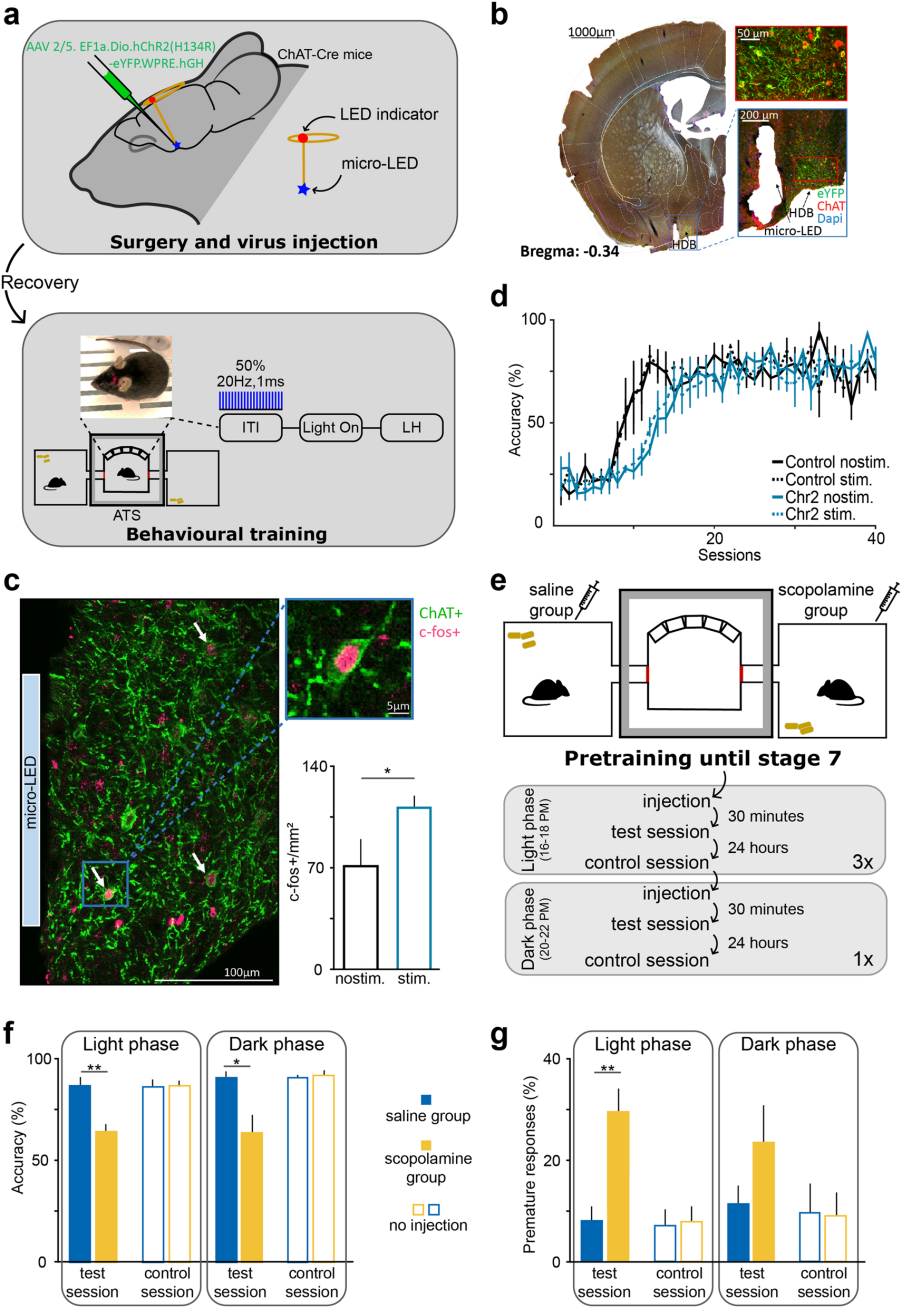
Optogenetic and pharmacological manipulations of the cholinergic system impaired performance. (**a**) Schematic representation of virus injection and implantation of a micro-LED for wireless optogenetic stimulation. Cholinergic neurons of the HDB area of the basal forebrain were stimulated with 1 ms pulses of blue light at 20 Hz in 50% of all trials in ChAT-Cre mice. Note the red indicator LED on the surface of the skull. (**b**) Confocal images of coronal sections showing viral infection in the HDB (green, eYFP) and a lesion indicating the location of the implant. Note that the implant was positioned lateral to the HDB, because the micro-LED was on the side of the holder. (**c**) Left, wireless optogenetic stimulation induced c-fos expression in the HDB of ChAT-ChR2 mice (green, eYFP; pink, c-fos; white arrows, c-fos+ cholinergic neurons; light blue rectangle, track of the micro-LED). The enlargement (top right) shows a c-fos-immunopositive channelrhodopsin-containing cholinergic neuron. Bottom right, wireless optogenetic stimulation significantly increased the number of c-fos-expressing neurons in the HDB. No stimulation, N = 3; stimulation, N = 4. (**d**) Accuracy as a function of sessions performed show that photostimulation delayed learning in ChR2-expressing mice compared to the controls (ChAT-Cre mice injected with a viral vector lacking the optogenetic actuator). Control, N = 4; ChR2, N = 4. (**e**) Schematic protocol of the pharmacology experiment. After reaching stage 7, one of the two mice trained in the same ATS received an ip. injection of scopolamine, while the other mouse was injected with saline. Injections were repeated three times with one day between the injections, allowing us to perform control sessions 24 h post injection. An additional experiment was performed when injections were administered in the dark phase of the animals. (**f**) Scopolamine had a negative effect on accuracy regardless whether it was injected in the light or in the dark phase of mice. No accuracy difference was seen in control sessions performed 24 h after the injections. Note that light phase results are the average of the 3 sessions, while dark phase injections were performed only once per animal. (**g**) Mice committed more premature responses after scopolamine injections during the light phase. Scopolamine-injected, N = 6; saline-injected, N = 6. *, *p* < 0.05; **, *p* < 0.01; one-way ANOVA; all values represent mean ± SEM.

We equipped the ATS with an apparatus for wireless optogenetic experiments and tested whether ATS training could be used in combination with optogenetic stimulations for behavioral manipulation studies. The photosensitive cation channel channelrhodopsin2 was expressed in the cholinergic neurons of the horizontal diagonal band (HDB), and activated with brief, 1 ms flashes of blue light at 20 Hz via unilaterally implanted micro-LEDs before the presentation of the light cues in 50% of the trials in a pseudorandomized manner (Fig. 7a–b). Photostimulation efficiently activated cholinergic neurons demonstrated by the expression of the activation marker immediate early gene c-fos in cholinergic neurons, at levels similar to what was reported previously^33^ (Fig. 7c). We did not find behavioral changes on stimulation trials within sessions, suggesting that the stimulation did not induce an acute trial-by-trial effect on sustained attention; however, when behavioral measures were compared as a function of sessions performed, control mice showed an earlier increase in accuracy, indicating faster learning (significant difference from session 1 to 24; group, F_1,12_ = 8.38, *p* = 0.013; time, F_23,276_ = 59.36, *p* < 0.0001; time x group, F_23,276_ = 4.78, *p* < 0.0001, repeated-measures ANOVA; Fig. 7d). This accuracy difference was accompanied by more premature response and fewer omissions in stimulated mice with a somewhat delayed time course (significant difference from session 13 to 36; premature, group, F_1,12_ = 4.50, *p* = 0.055; time, F_23,276_ = 13.94, *p* < 0.0001; time x group, F_23,276_ = 1.77, *p* = 0.018; omission, group, F_1,12_ = 6.42, *p* = 0.026; time, F_23,276_ = 8.55, *p* < 0.0001; time x group, F_23,276_ = 1.90, *p* = 0.0087, repeated-measures ANOVA; Supplementary Fig. S5).

Thus, cholinergic stimulation in the HDB delayed initial learning. A possible explanation for this effect is that artificial high-frequency cholinergic activation may interfere with precisely timed actions of the cholinergic system known to be important for learning and attention^34–36^. This experiment demonstrated that the ATS can be successfully combined with wireless optogenetic stimulation for testing behavioral manipulations.

Finally, to test whether the ATS can reproduce known pharmacological effects on 5CSRTT performance found using traditional manual training protocols, we tested the effect of the muscarinic antagonist scopolamine on behavior in the ATS. Scopolamine has been shown to reduce accuracy and increase premature responses in 5CSRTT^16,17,21,37^. Mice received scopolamine (N = 6) or saline injections (controls, N = 6) 30 min before training sessions. The effects of scopolamine injections were tested in both the light and dark phases of the animals (Fig. 7e; see also Methods). The first sets of injections, performed in the light phase, caused a drop in accuracy and an increase in premature responses (accuracy, group, F_1,10_ = 15.89, *p* = 0.0026; premature response, group, F_1,10_ = 15.92 , *p* = 0.0026; one-way ANOVA; Fig. 7f–g). The second set of injections, performed during the dark phase, had a significant impact on accuracy as well (accuracy, group, F_1,10_ = 9.46, *p* = 0.012). Interestingly, scopolamine-induced increase in premature responses was somewhat less pronounced in the dark phase than in the light phase of mice and hence only reached significance in the light phase injections (premature response, group, F_1,10_ = 2.28 , *p* = 0.16; one-way ANOVA; Fig. 7f–g). Mice allocated to scopolamine and saline groups did not show any behavioral differences in control sessions performed 24 h after the injections. Therefore, we replicated previously published pharmacological effects of scopolamine on 5CSRTT performance in mice trained in the ATS, suggesting that ATS-trained mice can serve to test behavioral effects similarly to previously used training protocols.

## Discussion

Rodents are capable of performing a large variety of cognitive tasks, which has rendered them a popular model for investigating how the brain controls behavior. However, rodents have almost exclusively been trained manually by human trainers, which limits training efficiency and may introduce covert biases. Here we presented a fully automated training system (ATS) for the 5-choice serial reaction time task, popular for investigating sensory detection, sustained attention and impulsivity^16,18,19,21,22^. The ATS, without PC, was estimated to cost $5,338 (Supplementary Table 1), which was approximately 25–29% of commercial solutions with comparable specifications. Mice engaged in training voluntarily on a regular schedule without any human interference throughout the entire training period. We showed that training in the automated system was more efficient and caused less stress to the animals. We equipped the training setup with wireless optogenetic stimulation and demonstrated the effect of both optogenetic and pharmacological manipulation on behavior. The ATS is modular, affordable, open source and can easily be adopted to a wide range of tasks.

Significant attempts have been made recently towards automated behavioral training^9–12,15,16,38,39^. One of the first automated training systems has been introduced in the Brody lab (Princeton University, US) for training rats on a flexible and expandable set of decision making tasks^9,40,41^. It solved training with no human interaction and served as a prototype of later systems. Nevertheless, it did not provide a comparison with traditional training methods and thus it left the question open whether the hard-to-formalize experimental decisions during training such as when to advance between training stages, when to terminate a session, whether and when to introduce training breaks, etc. can be automated without a compromise in training efficiency. Another milestone was marked by the Olvecky lab (Harvard University, US) that successfully combined automated training with automated recording in rats^8,10^, while the system was rather specific for that purpose. We have chosen the 5CSRTT, a popular rodent paradigm^20,21,42–44^, that has also been the subject of previous automation studies^16,17^. We have built on these earlier works by both providing an affordable, flexible, modular system as well as a systematic comparison with manual training in terms of training efficiency.

It was shown that training animals on the same operant task using either food or water reward had similar mild effects on animal wellbeing, while animals receiving water reward acquired the task faster, and were more motivated to work for reward^45^. In addition, fluid reward avoids chewing artifacts, making it easier to combine with neuronal recordings; therefore, we modified the 5CSRTT protocol to provide water reward instead of food pellets, and demonstrated fast training with water rewards. Finally, we scaled up training speed by attaching two home cages to one training chamber and demonstrated that it is possible to train two mice simultaneously in an alternating fashion.

Manual training on 5CSRTT may take 30–60 days or longer^23,46^. In contrast, we found that mice can be fully trained on 5CSRTT in the ATS in one weeks’ time. Nearly 40% of the auto-trained animals reached the highest stage 12 according to Bari’s training protocol^20^ after only one week of training, while all mice reached at least stage 6. In comparison, mice manually trained on the same task using previously described training protocols reached stage 2–3 when trained for equal number of days. In line with this, it took mice an average of 1.75 days to reach stage 3 in the ATS, but 6.46 days when trained manually; 3.94 days to reach stage 6 in the ATS, but 13.88 days to accomplish the same level in manual training, in agreement with an elegant recent 5CSRTT study^16^. The latter report by Remmelink and colleagues found that, similar to our ATS, mice needed significantly fewer days to finish a 5CSRTT protocol in a commercial automated training chamber, compared with manual training. However, the study did not include a side-by-side comparison of traditional and automated training. Therefore, to better understand the sources of training benefits, we compared training results (1) in an equal period of one week training, (2) in mice trained to the same stage (both 3 and 6) and (3) as a function of number of trials performed.

When we investigated the learning curves as a function of trials completed, manually and automatically trained mice did not show a large difference. Therefore, the main reason for the difference in training efficiency when considering the same one week training period was the higher number of trials mice completed in the ATS. This result held despite higher omission rate in the ATS, which could be a consequence of frequent access to water. However, when mice were trained to stage 3, which is relatively early in training in the ATS, manually trained mice showed somewhat higher accuracy (see Supplementary Fig. S1). This difference is consistent with established beneficial effects of sleep-related memory consolidation on behavioral performance^24^. When animals trained to stage 6 were compared, such differences were not observed (see ‘pretraining, last day’ in Fig. 5f–g), indicating that advantage from consolidation may disappear with the increasing number of training days. Nevertheless, as a net effect of these opposing trends, ATS-trained mice reached higher stages, including the highest stages 11–12, in a single week of training. Therefore, the automated training protocol may save significant amount of labor otherwise spent on manually training the animals and, at the same time, result in better trained mice in a substantially shorter time. We found that a stricter water schedule could increase the efficiency of manual training; however, these mice still reached lower training stage in one week of training time compared to ATS-trained animals. At the same time, we registered a tendency of higher weight loss in this cohort, raising concerns about increasing training efficiency by restricting access to water.

Manual protocols often train animals with pellet rewards, daily sessions, and might involve a handling period before training so that the animals get used to lab personnel. While scaling up manual training involves more human resources, increasing the number of mice trained in ATS (two mice per system in parallel with the present implementation) only requires increasing the number of ATS setups. Since these systems are affordable and open source (Supplementary Table 1)^47^, ATS provides a modular, readily scalable solution for mouse training. Moreover, we observed other benefits of automated training beyond cost efficiency. First, mice could be fully trained on 5-CSRTT within a week using the ATS, which has not been achieved in published literature using manual training. Second, ATS-training eliminated the potential of experimenter bias and a number of stressors, reflected in stress hormone measurement results (Fig. 6). Third, ATS cancels the effect of differences in mouse behavior due the time of day when training is performed^16,17^, since all mice undergo the same schedule. Fourth, animals in the ATS were able to continue training during weekends, conferences and even partial lockdowns with limited on-site personnel due to the recent COVID-19 pandemic.

In experiments where uniform behavioral performance is important, it is beneficial that the animals receive ‘pre-training’ before they undergo virus injection or implantation surgeries^48,49^. The surgery often affects the performance of the animals, likely due to a combination of factors such as lack of training during the recovery period, changes in head dimensions altering the access to important spaces of the training setup due to the implants, the need to retrain muscles due to muscle trauma and altered balance, and increased stress^50–52^. Previous studies have not addressed how these disturbances affect behavior in automated setups; therefore, we separately tested the effect of surgeries and training breaks on 5CSRTT performance in the ATS. We found that breaks both with and without surgery led to transient drops in performance. Such a performance lapse was not observed in manual training when mice were trained for equal number of days, possibly due to the more difficult task regime ATS-trained mice reached within this time. However, when mice in the manual and ATS groups were trained to stage 6, a training break caused by fiber implantation surgery resulted in a similar performance decrease in the manual setup. In all of these experiments, negative changes in behavioral measures were transient and reversed within one week while ATS-trained mice maintained their superior performance in terms of training stage; thus, training benefits of the ATS were maintained after the break. Similarly, transferring mice to the manual training setup after a training break only caused transient performance changes.

To further establish the assay’s practical use, we combined the automated training setup with wireless optogenetics^53^. This validation study enables an important technology to be immediately used in conjunction with our tool, broadening its scope of applications. Our design of two separate home cages connected with a single training chamber allowed automatic training of mice that express the optogenetic actuator^54^ in parallel with control mice in the same training box, minimizing the potential differences and uncontrolled factors between the two groups. It is important to remove potential subconscious biases in animal handling when performing optogenetic studies^2,4,5^, which we achieved in this arrangement. To measure events and control hardware with the timing precision necessary for concurrent electrophysiology, the ATS leverages Bpod, an open source behavior control system. Bpod’s finite state machine architecture enables simple reorganization of the task logic and flow of behavioral events in the ATS. The ATS system features five independent ports that can deliver either water reward or air-puff punishment with high temporal precision^1,11^. Since the system uses TTL pulses to synchronize optogenetic stimulation with the behavior controller, it is possible to precisely deliver photostimulation in any given task phase and part of the trial, allowing event or state specific manipulations^55–59^. To validate this application of our system, we showed that optogenetic stimulation of basal forebrain cholinergic neurons can interfere with quick learning of the 5CSRTT in the ATS, demonstrating feasibility of automated optogenetic manipulation experiments. We also demonstrated that injections of scopolamine, a muscarinic cholinergic antagonist, lead to an acute drop in accuracy as well as an increase in premature responses, as shown before in studies training mice on the 5CSRTT either manually or automatically^16,17,21,37^. Performance was fully recovered when tested 24 h after the injection.

Animal stress may impede learning and increase behavioral and neuronal variability, limiting the interpretation of certain metrics in behavioral neuroscience studies^60,61^. The increased variability may necessitate higher sample sizes, which, together with animal welfare concerns due to elevated stress, requires ethical considerations. We have partially eliminated important stressors during mouse training. Specifically, no human interaction was needed to carry out behavioral training in the ATS; additionally, mice were free to choose whether to engage in a given training session. Indeed, by measuring blood corticosterone levels, the main glucocorticoid stress hormone in rodents^27,28,30,31^, we found that training in the ATS caused significantly less stress to mice, which showed corticosterone levels similar to that of controls.

A single ATS setup is currently limited to training two mice in parallel. This may be improved in future iterations by using RFID chips and decoders, thus allowing the system to train multiple mice within the same ATS^11,12^. Another limitation is that while uni- or bilateral optogenetic manipulations can be performed wirelessly, this technology does not yet allow multi-target implants in mice due to size constraints. The ATS is not compatible with continuous tethering of mice; therefore, we transfer mice to manual training for tethered electrophysiology and optogenetic experiments. Nevertheless, we demonstrated that it is beneficial to pre-train mice in the ATS before implantation, and transferring from automated to manual training only introduces transient changes in performance measures, which are essentially the same when mice are pre-trained to the same stage manually.

The Automated Training System provides a fully automated, experimenter-free training environment. The animals each have the opportunity to begin a training session up to 12 times a day, which significantly accelerates learning. Participating in training sessions is not mandatory, and the amount of water consumed depends on the individual animals’ thirst and willingness to perform, which lead to reduced stress in the training environment. Mice trained in the ATS system had no difficulty switching to manual training while retaining their performance levels after few days of retraining. In the current implementation, two mice can be trained simultaneously on the 5CSRTT in one week, without any human interference. The system can readily be modified to train animals on a range of tasks, and we equipped the setup with wireless optogenetic stimulation to create an efficient, multi-purpose experimental tool.

## Methods

### Animals

Wild type male mice (N = 50, C57Bl/6J, over 6-weeks old) were used for the behavioral and pharmacological experiments and stress measurements; male homozygous ChAT-Cre mice (N = 31, over 6-weeks old) were used to test the effect of surgery and the wireless optogenetic stimulation. Male chat/cre_Δneo//Gt(ROSA)26Sor/CAG/LSL_chr2(H134R)/eyfp (ChAT-ChR2) mice were used for the c-fos experiment (N = 7). All experiments were approved by the Committee for Scientific Ethics of Animal Research of the National Food Chain Safety Office (PE/EA/675-4/2016, PE/EA/864-7/2019) and were performed according to the guidelines of the institutional ethical code and the Hungarian Act of Animal Care and Experimentation (1998; XXVIII, section 243/1998, renewed in 40/2013) in accordance with the European Directive 86/609/CEE and modified according to the Directives 2010/63/EU. Food was provided ad libitum (Special Diets Services VRF1), while water access was scheduled as described in details below. A small, 15 × 5 × 2 cm 3D-printed box filled with nesting material served as nest in the ATS. All animals were kept on a 12-h light–dark cycle. Light phase started at 7 am.

### Behavior setup

The ATS consisted of a central training chamber (16 × 16 × 10 cm) and two separate home cages, with controlled access to the central chamber. All chambers had grated floor with bedding underneath and were covered with a transparent plastic roof. Manual training was performed in an identical training chamber, but without the attached home cages. Manually trained animals were kept in standard mouse cages. The training chamber housed five water ports (Fig. 1, 2a; Sanworks, US). Each port was equipped with an infrared photogate to measure port entry, a white LED to display visual cues, and tubing for water delivery connected to separate water containers for each port via fast, high precision, low noise solenoid valves (LHDA1231115H, Lee Company, US). LED onsets, offsets and valve openings were controlled by port interface boards (#1004, Sanworks, US), connected to a Bpod open source behavior control system (Sanworks, US). The chambers were covered with soundproofing material^1^. A ‘house light’ LED was placed above the apparatus.

In the ATS, two 20 × 20 × 10 cm home cages were connected to the training chamber on each side through 10 × 5 × 4 cm tunnels. On both sides, the entrance to the training chamber was blocked by a motorized rodent gate, which was custom-designed and built from acrylic sheets and 3D-printed parts. The gate was powered by a commercially available servo motor (AX-12 W, Dynamixel). The home cages were equipped with passive infrared motion sensors (Panasonic EKMC series) attached to the home cage roof, directly above the tunnel entrance. Opening and closing of the gates in response to motion detection was controlled by custom firmware on an Arduino Leonardo (Fig. 1b–c). We set up a 24-h surveillance system with web cameras and red lighting for the night period (Fig. 1a–b). The cameras were accessed remotely to periodically check the operation of the ATS. Behavior control code was developed in Matlab and Arduino languages. See Supplementary Table 1 for Bill of Materials.

### Wireless optogenetic stimulation

The ATS was combined with a commercial wireless optogenetic stimulation system (NeuroLux, Fig. 1c). We wrapped the coil of the wireless system around the training chamber, which then created an electromagnetic field that powered an implanted micro-LED. The LED was emitting blue light (470 nm) upon induction through the coil. The optogenetic stimulation system allowed for precise, automated control of LED onsets an offsets by TTL signals^53^. Implanted mice were photostimulated with 1 ms, 8 W light pulses at 20 Hz during the inter-trial intervals in half of the trials, allocated in a pseudorandomized order. Mice used for c-fos expression measurements were stimulated with the same parameters in their home cages for 15 min (3 s stimulation, 6 s pause).

### Training protocol

Mice were randomly assigned to experimental groups. Water reward was used for motivation: animals undergoing manual training (wild type, N = 16) were subjected to a standard water restriction schedule, where they received water according to task performance during a 30-min training session daily and additional free water for 2 h/day, at least 2 h after their last training session (from 2 to 4 pm). A group of mice (ChAT-Cre, N = 6) trained in the manual setup were kept on a different water restriction schedule. These animals were supplemented to a total amount of 1.8 ml at 4 pm daily, to match the daily average water intake of mice trained in the ATS. Animals trained in the ATS (wild type, N = 16; ChAT-Cre, N = 25) received their entire water intake from the task in the training chamber, accessed regularly every two hours for a 15-min self-training sessions (Fig. 2a–b). All ports of the training chamber were calibrated to provide the same amount of water in each port, and delivered distilled water to avoid clogging of the tubing and valves; therefore, we placed a piece of mineral stone (Panzi, Hungary) as ion supplement in the home cages of the ATS. Weight of the animals was regularly monitored.

During 5CSRTT, animals had to repeatedly detect flashes of light above one of the five ports presented in a pseudorandom order and report the detection by performing a nose poke in the respective water port. Upon correct reporting, 4–6 µl of water was delivered from the port as reward. Every session started with free access to 10–20 µl water from each port (in the manual training group, only in stage 1). Each trial started with an intertrial interval (ITI), in which poking in the ports was prohibited. After the ITI, one of the ports was illuminated (Light On). The animal had to poke its snout into the illuminated port during ‘Light On’ or a short time period after that (limited hold, LH), in order to get the water reward. A poke during the ITI (premature response), in the incorrect port during Light On or LH (incorrect answer), or missing the periods allotted for nose poke (omission) resulted in a 5-s timeout, during which the house light was turned off. Each trial ended with either reward or a time-out punishment (Fig. 2c).

The length of the ITI, Light On and LH varied across training stages to enable a progressive increase of difficulty. Mice were allowed to switch stages during a session in case they passed predefined criteria (Table 1). Reward amount was set to 6 µl in stage 1, 5 µl in stage 2 and 4 µl in all subsequent stages. From stage 3, we randomized the duration of the ITI among 3, 4 or 5 s to increase attentional demand of the task.

A group of mice (N = 5) was pretrained manually, and a separate group was pretrained in the ATS (N = 14) until they reached stage 6, then trained for an additional day (also included in manually and ATS-trained groups above). All of these mice underwent the same surgical implantation protocol (see below) and were subsequently trained in the manual setup at stage 6 with the standard water schedule after a 3-week recovery period. Before each session, optic patch cables were connected to the implanted optic fibers to mimic optogenetic experiments; however, photostimulation was not performed in these mice.

A group of mice (wild type, N = 6) did not receive any training and served as controls for stress hormone measurements.

### Surgery

Mice were anesthetized with an intraperitoneal injection of a ketamine-xylazine combination (25 mg/kg xylazine and 125 mg/kg ketamine dissolved in 0.9% saline) and placed in a stereotaxic frame (Kopf Instruments, US). Local anesthetic (Lidocaine, Egis, Hungary) was applied subcutaneously and the eyes were protected by ophthalmic lubricant (Corneregel, Benu, Hungary). The skull was cleared and an opening was drilled above the horizontal diagonal band of Broca (HDB), a major hub of the central cholinergic system implicated in learning and attention^35,62^. A pipette pulled from borosilicate glass capillary was lowered into the target area and an adeno-associated viral vector (control virus: AAV 2/5. EF1a.Dio.eYFP.WPRE.hGH used to test the compatibility of ATS with wireless optogenetics and for control animals in the optogenetic stimulation protocols; ChR2 virus: AAV 2/5. EF1a.Dio. hChR2(H134R).eYFP.WPRE.hGH used to activate cholinergic neurons in the wireless optogenetic experiments; ArchT virus: AAV2/5_CAG_Flex_ArchT_GFP used to mimic wired optogenetic experiments) was injected (300 nl to AP, + 0.75; MD, + /− 0.6; DV, − 5.0 and − 4.7 mm) unilaterally (in the case of control and ChR2 viruses) or bilaterally (in the case of ArchT virus) into the HDB. Mice were either implanted with a wireless implant consisting of a needle-shaped holder, a micro-LED on its side (illuminating the brain area lateral to it) and an optogenetic sensing module (NeuroLux, US), or optic fibers (200 µm core diameter, LC-LC connector) for wired optogenetic experiments. The wireless implant was lowered into the HDB unilaterally (AP, + 0.75; MD, + /− 1; DV, − 5.5 mm). We secured the ring-shaped optogenetic sensing module to the surface of the skull with tissue glue (Vetbond, 3 M, US). The needle that held the LED was cemented to the skull with dental cement (Paladur, Dentaltix, Italy). The optic fibers for wired experiments were placed into the HDB bilaterally (6°, AP, + 0.75; MD, + /− 1.07; DV, − 4.4) and the implants were secured to the skull with dental cement as above. The skin was sutured and antibiotic cream (Baneocin, Medigen, Hungary) was applied on to the surgical wound. The animal was placed on a heating pad for recovery. Buprenorphine was used for post-operative analgesia (Bupaq, 0.3 mg/ml; 528 Richter Pharma AG, Wels, Austria). A rest period of 2–3-weeks was allowed for full recovery, after which the experimental protocols were initiated.

### Immunohistochemistry

After termination of the wireless optogenetic experiment, mice were deeply anesthetized with ketamine-xylazine (see above). Mice were perfused transcardially with 0.1 M phosphate buffered saline (PBS) for 3 min, then with 4% paraformaldehyde (PFA) in PBS for 20 min. The implants were carefully removed, and brains were post-fixed for 24 h in PFA at + 4 °C. 50-μm-thick coronal sections were cut (Vibratome VT1200S, Leica, Wetzlar, Germany). To facilitate the identification of the HDB and the position of the implant, immunofluorescent staining of the cholinergic cells was carried out. Primary antibody was diluted in Tris-buffered saline (TBS) (Rabbit-anti-Choline Acetyltransferase, 1:1000, SySy, CatNo: A-11039; Goettingen, Germany) and were incubated for 2 days. After washing, sections were incubated in secondary antibody solution overnight (Alexa594-conjugated Donkey-anti-Rabbit, 1:500, Jackson ImmunoResearch, CatNo: 711-585-152; Cambridge House, UK). As the last step, sections were stained with 10 mg/ml 4′-6-diamidino-2-phenylindole dye (DAPI, Dihydrochloride, Merck Millipore, Burlington, MA, USA). Sections were mounted on microscope slides, covered in mounting medium (Vectashield, Vector Labs, Burlingame, CA, USA) and examined with a confocal microscope (Nikon Eclipse Ni microscope, Nikon Instruments, Melville, NY, USA).

### Measurements of c-fos expression

Chat/cre_Δneo//Gt(ROSA)26Sor/CAG/LSL_chr2(H134R)/eyfp (ChAT-ChR2) mice were used for measurements of c-fos expression. Optogenetic stimulation was performed as described above. Ninety minutes after the wireless optogenetic stimulation in the homecage, animals were anaesthetized with ketamine-xylazine and perfused transcardially (see above). The c-fos protein was labeled with a rabbit polyclonal anti-c-fos antibody (1:5000, 226 003, Synaptic Systems) and tissue sections were also immunostained with a chicken polyclonal anti-GFP antibody (1:2000, A10262, TermoFisher) to amplify the signal of the ChR2-containing neurons. Sections were incubated for 72 h in primary antibody solutions that contained 2% NDS and 0.1% Triton in PBS. This step was followed by 2 h incubation with secondary antibodies (Alexa Fluor 488-labeled goat anti-rabbit, 1:1000, A11039, Life Technologies Alexa Fluor 647-labeled donkey anti-chicken, 1:1000, 711-605-152, Jackson Laboratories). The place of the LED was defined according to the mouse brain atlas of Paxinos and Franklin^63^. To quantify c-fos expression, fluoromicrographs were taken (Nikon Eclipse Ni microscope, Nikon Instruments, 1024 × 1024 pixels/image) using a 20 × magnification lens. Uniform laser intensities and global thresholds were used for the measurements among slices. c-fos expressing GFP-positive neurons were counted in the area of the HDB in front of the LED in each section, allowing us to analyze 3–4 50-µm-thick sections given the dimensions of the LED (widths, 220 µm). The number of c-fos-positive cells was normalized by the area of the HDB in each section.

### Pharmacology

Mice were pretrained and kept at stage 7 in the ATS. To test the effect of scopolamine on 5CSRTT performance in the light phase, mice were put onto a nearby desk for injections at a fixed time of day (4:30 pm or 5:30 pm). Mice were injected with scopolamine (scopolamine hydrobromide dissolved in saline, 1414, Tocris, 1 mg/kg introduced intraperitoneally within 24 h of the animal reaching stage 7), or saline for the control animals, then returned to the training setup immediately. The test session started 30 min after the injection. No injection was performed on the following day, allowing for performing control sessions 24 h post injection. Injections in the dark phase were performed at 8:30 pm or 9:30 pm the same way as the daytime injections in a darkened room (minimal light was necessary for performing the injections). Injections in the light phase were repeated 3 times for each mouse. Injections in the dark phase were performed only once for each mouse.

### Measuring the stress level of the animals

To measure acute stress of the animals caused by training (and handling in case of manually trained animals), blood samples were collected after their last training session. On the 7th day (9 am to 12 pm), manually trained animals were placed in their home cages after training, for 10 min. After training at matching time of the day, the animals in the ATS were allowed to return to their home cages within the system for 10 min. Water consumption was similar in the two groups during the last training sessions. After the 10 min rest, mice were transferred to a separate room. For corticosterone level measurements, blood samples were collected during decapitation in ice-cold plastic tubes, centrifuged and the serum was separated and stored at − 20 °C until analysis. Corticosterone was measured in 10 μl unextracted serum or undiluted medium by a radioimmunoassay (RIA) using a specific antibody developed in our institute as described earlier^64,65^. Samples from each experiment were measured in a single RIA (intra-assay coefficient of variation, 7.5%). We compared data after one week of training in three groups (control, N = 6; manually trained, N = 6; ATS-trained, N = 6). In the control group (N = 6), mice had food and water available ad libitum and were not handled. The behavioral data of these animals were included in Figs. 3, 4, 5.

### Statistics

Behavioral performance was analyzed by custom-written open source code in Matlab 2016b (MathWorks, US) available at https://github.com/hangyabalazs/ATS. Performance parameters were defined as follows: accuracy, correct responses/(correct + incorrect responses) * 100; premature responses, prematurely terminated trials/all trials * 100; omission responses, omitted/(correct + incorrect + omitted trials) * 100; reaction time, time between Light On and correct response in seconds^23^. Daily performance metrics for animals in the ATS were calculated as the average of all sessions of the day. Statistical analysis was carried out using the STATISTICA 13.4 software (TIBCO, US). Group differences were assessed by one-way, repeated measures ANOVA. Newman–Keuls post hoc tests were performed after ANOVA if the main effects were significant. Wilcoxon singed-rank test and Mann–Whitney U-test were used for non-parametric comparison of central tendencies between two paired or unpaired distributions, respectively. Data are presented in the figures as mean ± standard error. Differences were considered significant at *p* < 0.05.

## Supplementary information

Supplementary Information 1.

## Data Availability

The datasets generated and/or analysed during the current study are available from the corresponding author on reasonable request.

## Code availability

Code for hardware control and behavioural data analysis can be downloaded from https://github.com/sanworks/Pipeline_Gate and https://github.com/hangyabalazs/ATS.
