# Supplementary material for: Efficient training of mice on the 5-choice serial reaction time task in an automated rodent training system
Source: Sci Rep. 2020 Dec 21;10:22362. doi: 10.1038/s41598-020-79290-2 (PMC7752912; doi:10.1038/s41598-020-79290-2)
Supplement: Supplementary file 1 — Supplementary Information 1. [file 41598_2020_79290_MOESM1_ESM.pdf]

Supplementary Material

Efficient training of mice on the 5-choice serial reaction time task in an automated rodent training system

Eszter Birtalan[1], Anita Bánhidi[1], Joshua I. Sanders[2], Diána Balázsfi[1,*], Balázs Hangya[1,*]

[1]Lendület Laboratory of Systems Neuroscience, Institute of Experimental Medicine, Budapest, Hungary

[2]Sanworks LLC, Rochester, NY, United States

*correspondence: balazsfi.diana@koki.mta.hu, hangya.balazs@koki.mta.hu

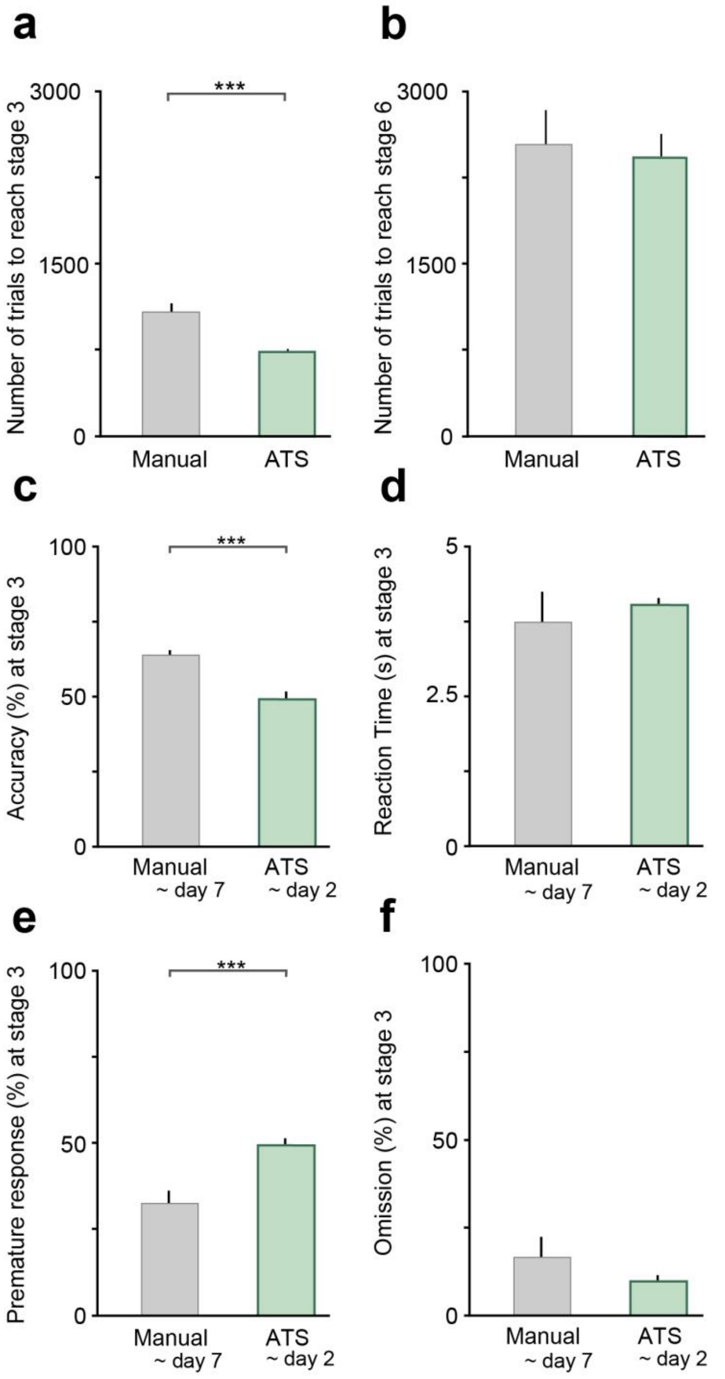

Supplementary Figure S1. Number of trials to reach performance criterion and comparison of stage 3 performance. (a-b) Number of trials needed to reach stage 3 (a) or stage 6 (b) in manual and ATS setups. (c-f) Behavior measures during sessions at stage 3 (manual: approx. day 7; ATS: approx. day 2). Manually trained mice performed with higher accuracy (c), but with similar reaction times (d). Manually trained mice performed fewer premature responses (e and similar number of omissions (f). ***, $p < 0.001$; one-way ANOVA; manual, N = 13; ATS, N = 16.

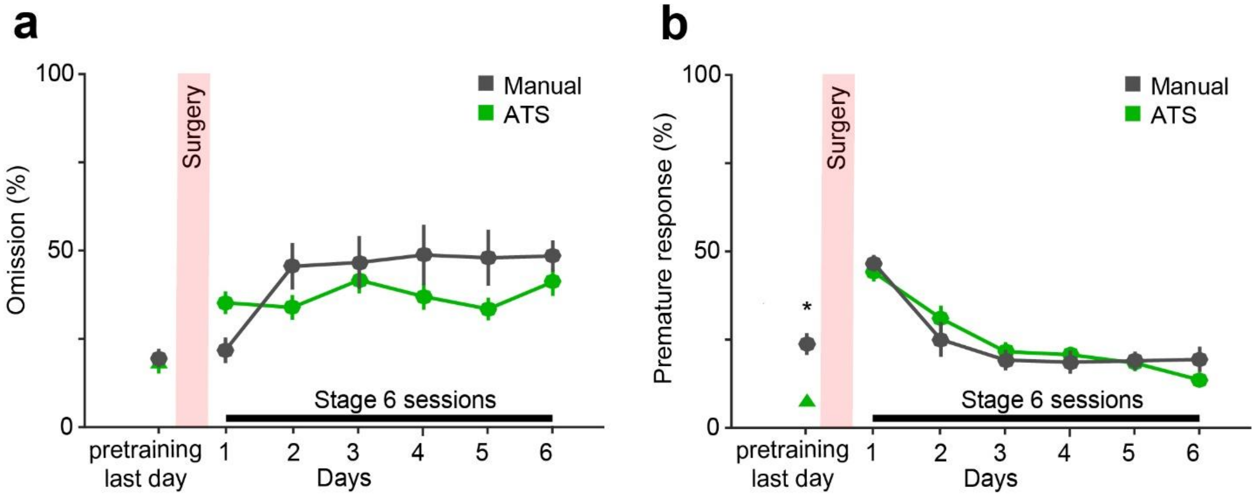

Supplementary Figure S2. Time course of premature and omitted responses after surgery. (a) Omissions did not differ between the groups. (b) Animals pre-trained manually performed more premature responses on the last day of pretraining. No other difference was found between the two groups. *, $p < 0.05$; one-way ANOVA; manual, N = 5; ATS, N = 14; all values represent mean ± SEM.

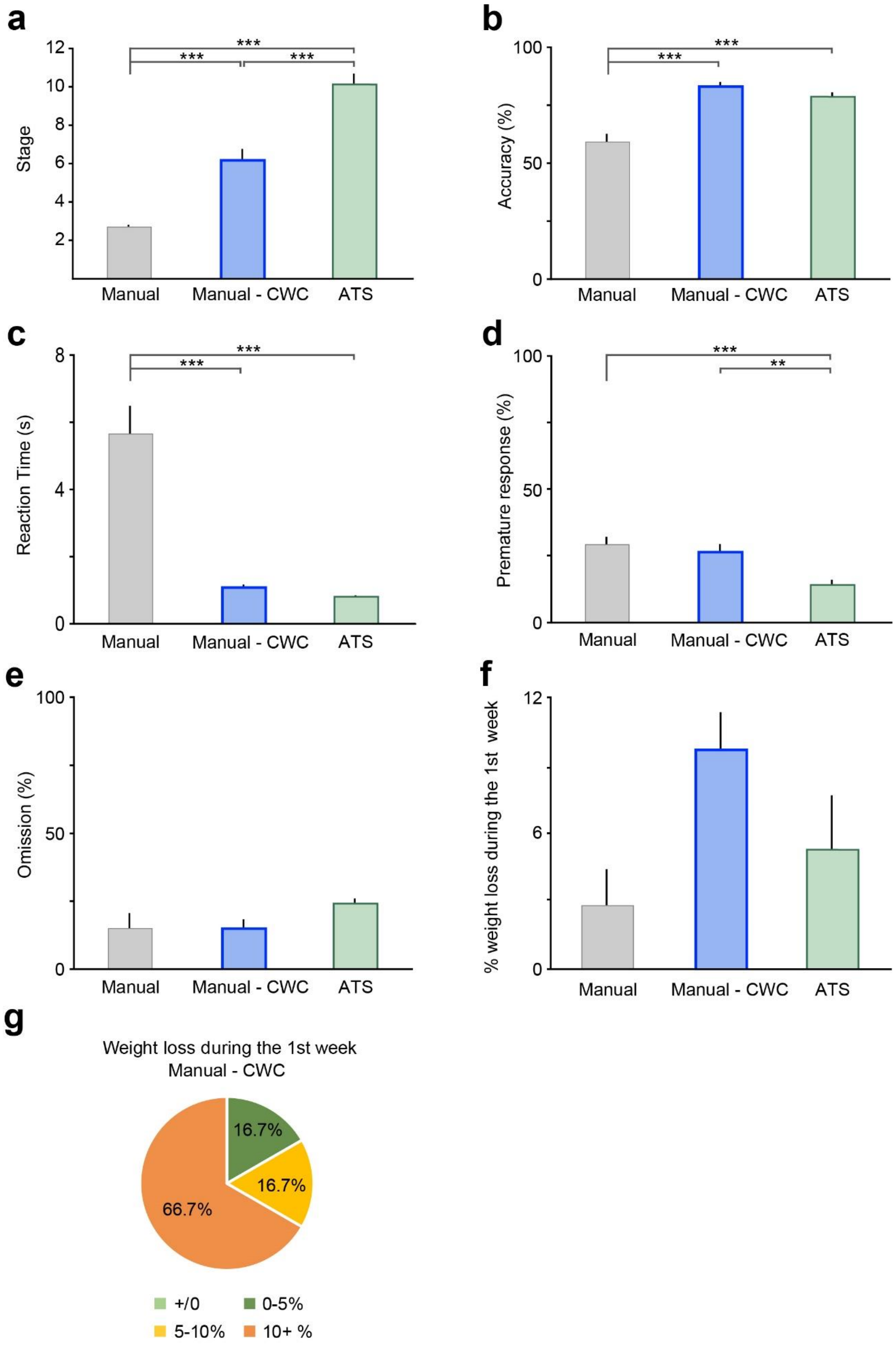

a
Stage
12
10
8
6
4
2
***
***
***
Manual
Manual - CWC
ATS
b
Accuracy (%)
100
50
0
***
***
Manual
Manual - CWC
ATS
c
Reaction Time (s)
8
4
0
***
***
Manual
Manual - CWC
ATS
d
Premature response (%)
100
50
0
***
**
Manual
Manual - CWC
ATS
e
Omission (%)
100
50
0
Manual
Manual - CWC
ATS
f
% weight loss during the 1st week
12
6
0
Manual
Manual - CWC
ATS
g
Weight loss during the 1st week
Manual - CWC
16.7%
16.7%
66.7%
+/0
0-5%
5-10%
10+ %

Supplementary Figure S3. Comparison of one week of manual training with different water restriction schedules and ATS training. (a-e) Performance measures during the 7th day of training. (a) Mice trained with a controlled water consumption (CWC) schedule reached higher stages than the manual group, but lower stages than the ATS-trained mice. (b) There was no difference in accuracy between the manual-CWC and ATS groups, but both performed with higher accuracy than the manual group. (c) The manual-CWC group showed reaction times similar to ATS-trained mice. (d) The manual-CWC group showed a number of premature responses comparable to the manual group, both higher than ATS-trained mice. (e) There was no difference in omissions between the groups. (f) Comparison of body weight changes between training day 1 and 7. Mice in the manual-CWC group showed a tendency to lose more weight. (g) Distribution of % weight loss of mice in the manual-CWC group by the end of the one-week training period. More than 50% of the animals of this group lost more than 10% of their body weight. ** $p < 0.01$, *** $p < 0.001$; one-way ANOVA; manual, N = 16; manual-CWC, N = 6; ATS, N = 16.

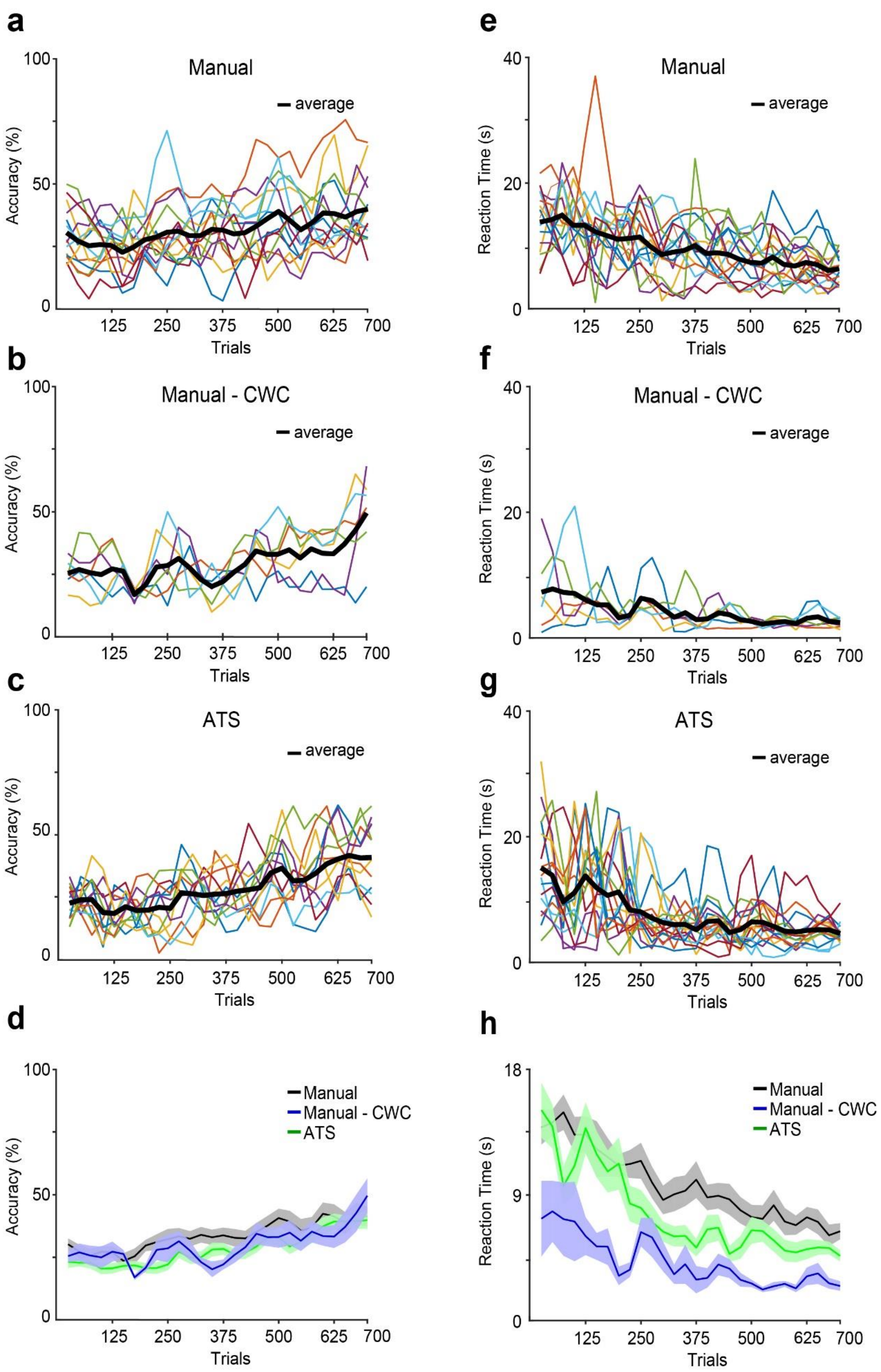
a
Manual
average
Accuracy (%)
Trials
b
Manual - CWC
average
Accuracy (%)
Trials
c
ATS
average
Accuracy (%)
Trials
d
Manual
Manual - CWC
ATS
Accuracy (%)
Trials
e
Manual
average
Reaction Time (s)
Trials
f
Manual - CWC
average
Reaction Time (s)
Trials
g
ATS
average
Reaction Time (s)
Trials
h
Manual
Manual - CWC
ATS
Reaction Time (s)
Trials

Supplementary Figure S4. Accuracy and reaction time during the first 700 trials of training. (a) Accuracy calculated for the first 700 trials of training (in 50-trial sliding windows with 50% overlap) for manually trained mice. Colored lines, individual mice; black line, average. (b) Same as in (a) but for the manual-CWC group. (c) Same as in (a) but for the ATS-trained mice. (d) Average accuracy in the first 700 trials in the manual (grey), manual-CWC (blue) and ATS groups (green); lines and error shades represent mean ± SEM. (e) Reaction time calculated for the first 700 trials of training (in 50-trial sliding windows with 50% overlap) for manually trained mice. Colored lines, individual mice; black line, average. (f) Same as in (e) but for the manual-CWC group. (g) Same as in (e) but for the ATS-trained mice. (h) Average reaction time in the first 700 trials in the manual (grey), manual-CWC (blue) and ATS groups (green); lines and error shades represent mean ± SEM.; manual, N = 16; manual-CWC, N = 6; ATS, N = 16.

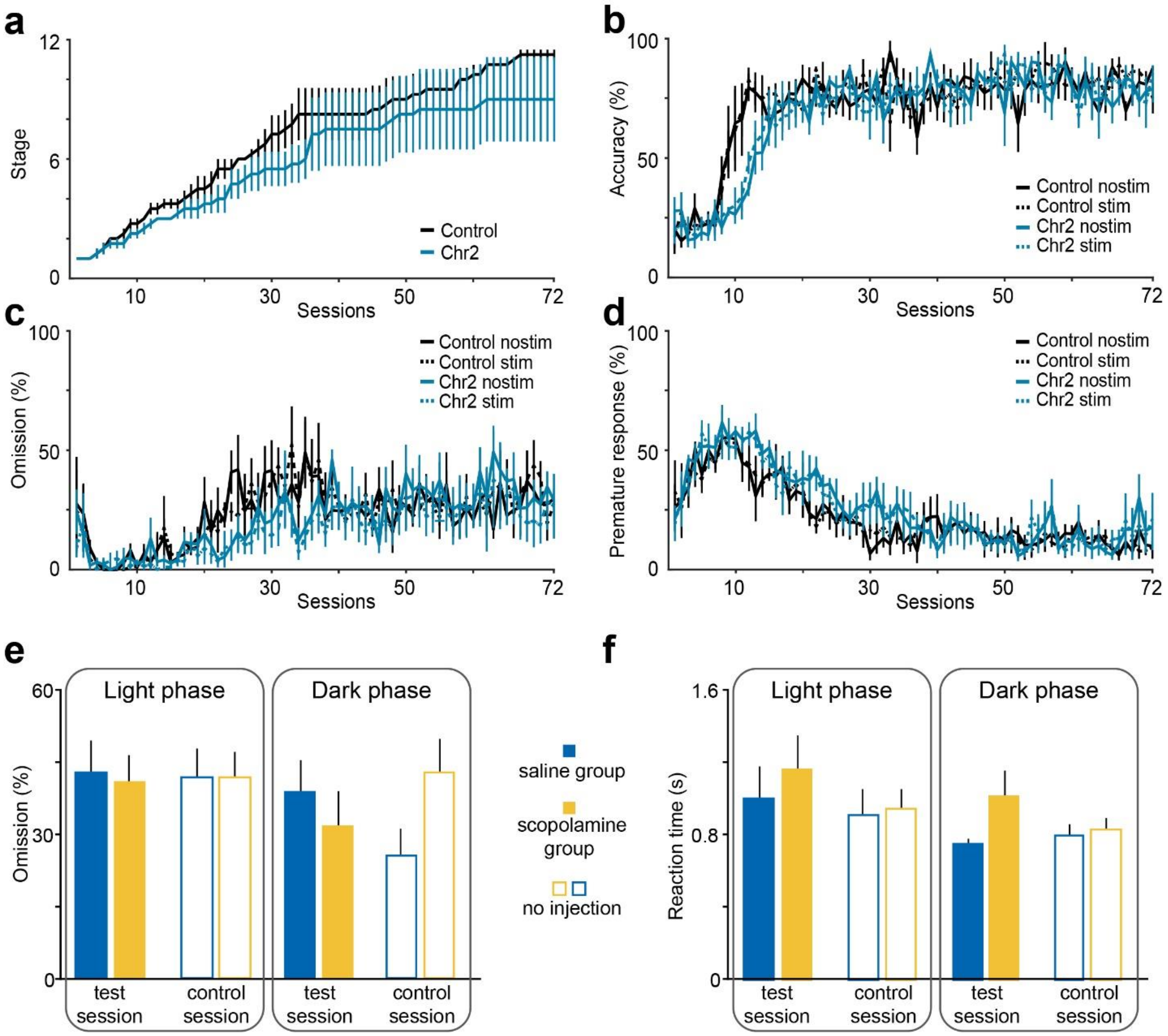

Supplementary Figure S5. Wireless optogenetic stimulation of basal forebrain cholinergic neurons. (a) Cholinergic neurons of the HDB area were stimulated during training (see Methods). Control mice showed a tendency to reach higher stages as a function of sessions completed than ChR2-expressing mice. (b) Stimulated mice showed slower initial learning. This difference disappeared during later training as mice reached the same asymptotic accuracy. (c-d) ChR2-expressing animals performed fewer omissions (c) and more premature responses (d) than mice in the control group. Control, N = 4; Chr2, N = 4. (e-f) Scopolamine injections had no significant effect on the number of omissions and on reaction time, regardless whether they were performed in the light or in the dark phase of the animals. Note that results of the light phase are the average of the 3 sessions after the 3 injections, while dark phase injections were performed only once per animal. Scopolamine-injected, N = 6; saline-injected, N = 6. All values represent mean ± SEM.

Supplementary Table 1

	Vendor	Amnt	Price
Automated 5CSRTT setup			EUR
Custom design of apparatus for 5-port serial reaction time task	Sanworks - by request	1	785
5-port serial reaction time apparatus – laser cut parts	Sanworks - by request	1	231
5-port serial reaction time apparatus – 3D printed parts	Sanworks - by request	1	208
White LED diode (TC-LED 3MM diffuse white 700MCD)	Conrad Electronic - 1573704	1	0,01
Mouse port assembly	Sanworks - 1009	5	1132
Pipeline motorized gate and USB gate controller	Sanworks - by request	2	642
Pipeline enrichment box with infrared motion sensor	Sanworks - by request	2	734
Pipeline wall for mouse box	Sanworks - by request	2	32
Logitech webcamera C525	Logitech - 960-001064	3	188
Hub (Renkforce RF-4847586 USB 3.0 hub)	Conrad Electronic - 1615862	1	40
Red LED strip (SMD 3528 LED, 6-7 lm/LED)	LED-plaza.hu - 3528-60 5V red	1 m	3
Behaviour control unit			
RJ45 CAT 5e U/UTP network cable	Conrad Electronic - 990377	5	9
BPOD State Machine r1	Sanworks - 1027	1	710
USB 2.0 cable (USB A-micro B)	Conrad Electronic - 1556351	3	12
Computer			
Dell Optiplex 5060 SFF-nVidiaPCIe2GB Black Windows 10 Pro, Intel Core i7-8700, Intel Chipset, 8GB, 2666MHz, Gigabit, 2.0, SSD 512GB, nVidia PCIE (GPU:1227/1468 | 1252/1506MHz, RAM:6008MHz, 2048MB, DDR5, 64bit, 1xDVI, 1xHDMI, 2xDisplayport , LP+Intel HD Graphics 630), 4xUSB2.0, 5xUSB3.1, 1xUSB Type-C, 92,6x290x292mm, keyboard, mouse	Smart Info	1	700
Dell 24" U241EXH LED Monitor	Smart Info	1	250
Water delivery			
Nalgene™ 180 Clear Plastic PVC Metric Tubing - 2mm id (0.078 in) 4mm od (0.039 in)	Thermo Scientific – 8001-0204	25 ft	14
60ml syringe with Luer-Lok tips	BD Plastipak - 309653	5	3

		EUR
TOTAL	without PC	4743
	with PC	5693

Supplementary Table 1. Bill of materials for the ATS.
